# Pleiotropic Benefit of Monomeric and Oligomeric Flavanols on Vascular Health - A Randomized Controlled Clinical Pilot Study

**DOI:** 10.1371/journal.pone.0028460

**Published:** 2011-12-08

**Authors:** Antje R. Weseler, Erik J. B. Ruijters, Marie-José Drittij-Reijnders, Koen D. Reesink, Guido R. M. M. Haenen, Aalt Bast

**Affiliations:** 1 Department of Toxicology, Maastricht University, Maastricht, The Netherlands; 2 Department of Biomedical Engineering, Maastricht University, Maastricht, The Netherlands; Brigham & Women's Hospital - Harvard Medical School, United States of America

## Abstract

**Background:**

Cardiovascular diseases are expanding to a major social-economic burden in the Western World and undermine man's deep desire for healthy ageing. Epidemiological studies suggest that flavanol-rich foods (e.g. grapes, wine, chocolate) sustain cardiovascular health. For an evidenced-based application, however, sound clinical data on their efficacy are strongly demanded.

**Methods:**

In a double-blind, randomized, placebo-controlled intervention study we supplemented 28 male smokers with 200 mg per day of monomeric and oligomeric flavanols (MOF) from grape seeds. At baseline, after 4 and 8 weeks we measured macro- and microvascular function and a cluster of systemic biomarkers for major pathological processes occurring in the vasculature: disturbances in lipid metabolism and cellular redox balance, and activation of inflammatory cells and platelets.

**Results:**

In the MOF group serum total cholesterol and LDL decreased significantly (*P*≤0.05) by 5% (n = 11) and 7% (n = 9), respectively in volunteers with elevated baseline levels. Additionally, after 8 weeks the ratio of glutathione to glutathione disulphide in erythrocytes rose from baseline by 22% (n = 15, *P*<0.05) in MOF supplemented subjects. We also observed that MOF supplementation exerts anti-inflammatory effects in blood towards ex vivo added bacterial endotoxin and significantly reduces expression of inflammatory genes in leukocytes. Conversely, alterations in macro- and microvascular function, platelet aggregation, plasma levels of nitric oxide surrogates, endothelin-1, C-reactive protein, fibrinogen, prostaglandin F2alpha, plasma antioxidant capacity and gene expression levels of antioxidant defense enzymes did not reach statistical significance after 8 weeks MOF supplementation. However, integrating all measured effects into a global, so-called vascular health index revealed a significant improvement of overall vascular health by MOF compared to placebo (*P*≤0.05).

**Conclusion:**

Our integrative multi-biomarker approach unveiled the pleiotropic vascular health benefit of an 8 weeks supplementation with 200 mg/d MOF in humans.

**Trial Registration:**

ClinicalTrials.gov NCT00742287

## Introduction

Cardiovascular diseases (CVD) are expanding to a major social-economic burden in the Western World [1], [2]. Despite a diverse disease pattern in advanced states, the early onset of CVD is characterized by the occurrence of several general pathophysiological mechanisms. Persistently increased levels of inflammation and oxidative stress, elevated serum lipid levels as well as the development of a prothrombotic state crucially contribute to the occurrence of an impaired vascular function [3]. Chronic cigarette smoking is one of the lifestyle factors that essentially fuels and exaggerates these processes. It is well established that cigarette smoke contains large quantities of free radicals and pro-oxidant compounds [4] and additionally increases endogenous free radical production by the activation of macrophages and neutrophils, uncoupling endothelial nitric oxide synthase (eNOS) and mitochondrial transport chain [5]–[7]. As a consequence markers of oxidative stress and inflammation are significantly elevated in the systemic circulation of smokers compared to non-smokers [8]–[12]. Heightened serum lipid levels and the activation of platelets further promote the manifestation of a proatherogenic state in smokers [13], [14] and contribute to the development of endothelial dysfunction in coronary and peripheral conductance and resistance vessels [15], [16]. Furthermore, dietary antioxidants like vitamin C have been shown to improve or even reverse proatherogenic, proinflammatory and prothrombotic conditions in smokers [17], [18].

In search of strategies which are able to sustain long-term cardiovascular health and are easily implementable in peoples' everyday life, dietary supplementation may indeed offer valuable opportunities. Epidemiological studies performed over the past decades underpin that a traditional Mediterranean diet reduces the risk of CVD [19], [20]. Since polyphenols, in particular monomeric and oligomeric flavan-3-ols (MOF) consisting of up to 5 flavanol units, are an integral part of this diet, these compounds have been widely studied *in vitro* and *in vivo* in order to elucidate their mechanism of action to prevent CVD [21]–[24]. The seeds of grapes (*Vitis vinifera* L.) are in particular rich in MOF. Since grape seed extracts consist of different mixtures of MOF and other polyphenols, a thorough analysis of their composition is indispensable for characterizing the active principle. Moreover, various MOF are supposed to modulate diverse (patho)physiological processes, which requires the assessments of multiple clinical endpoints. Beyond such multifaceted mechanisms of action, nutrients, in contrast to drugs, are known to modulate human's body function in a subtle manner. As a consequence, the classical way of proving clinical effectiveness by the assessment of a single primary end point does not adequately reflect the mode of action of these compounds and frequently fails to unveil significant effects [25]. Therefore, the present pilot study aimed to investigate the pleiotropic effects of a relatively low dose supplementation with a well-characterized MOF composition from grape seeds in the human vasculature in a holistic manner. The meticulous selection of outcome parameters led to a spectrum of makers that comprised established as well as novel cardiovascular risk factors and systemic biomarkers reflecting the most essential pathomechanisms in the human vasculature on a functional and (sub)cellular level. The integration of the changes in these biomarkers into a vascular health index enabled us to demonstrate the beneficial effects of MOF on vascular health in general. This is the first trial that applies an integrative biomarker approach in order to determine the health effects of a dietary supplementation in the human vasculature.

## Methods

The protocol for this trial and CONSORT checklist are available as supporting information; see Checklist S1 and Protocol S1.

### Subjects and study design

The study was designed as a randomized, placebo-controlled, double-blind trial and conducted at the Maastricht University and Academic Hospital Maastricht, The Netherlands. Non-obese men were eligible when they were between 30 and 60 years old and smoked ≥10 cigarettes per day for at least 5 years. Exclusion criteria were a history or presence of any metabolic, cardiovascular and/or malignant disease, excessive consumption of alcohol (>28 consumptions, i.e. approximately 250 g/week), a vegetarian/vegan life style, medically prescribed diet or slimming diet and the use of any supplement and functional food containing vitamins, antioxidants and/or polyphenolic compounds for 4 weeks before and during the study. Thirty-three eligible subjects were included and randomized in the study (Figure S1). Five subjects dropped out either before or after the 1^st^ study visit due to personal reasons which were not related to the study.

All subjects gave their written informed consent prior to their participation. The study was approved by the Medical Ethical Committee of the Maastricht University and Academic Hospital Maastricht, The Netherlands and conducted in accordance with the World Medical Association Declaration of Helsinki of 1975 as revised in 2008.

Subjects were randomly assigned to one of the two test groups under taking into account that the groups became balanced with respect to the number of cigarettes smoked per day.

The two parallel supplementation regimes consisted of capsules containing either 100 mg MOF from *Vitis vinifera* L. seeds (MASQUELIER's® Original OPCs) or an equivalent amount of microcrystalline cellulose (placebo). The composition of the verum capsules regarding its standardized MOF content is shown in Table 1. The MOF and the placebo material were provided by International Nutrition Company (INC) BV, Loosdrecht, The Netherlands in indistinguishable opaque capsules which were packaged in blisters and boxes labeled with the treatment code. Subjects and investigators were blinded for the treatment code until data analysis was completed. The subjects were asked to daily take 2 capsules with a glass of water in the morning directly before breakfast and to note the time of intake in their study diary. In addition, subjects were instructed not to change their daily eating, smoking and life style habits and to record all potential deviations in their study diary on a daily base. Every 2 weeks subjects were invited for a control visit at the investigational site in order to control their well-being and the occurrence of potential adverse events. At these occasions a comparable number of subjects of both test groups reported discomfort from common cold, headache, nausea, and shoulder and ankle injury during the 8 study weeks. All these events were classified by the subjects as mild and were not related to the intervention. The intake of the test capsules was checked based on the entries in the study diary and the blisters returned. These controls revealed full intake compliance in both test groups (median, 100%).

**Table 1:** Composition of monomeric and oligomeric flavanols isolated from grape (*Vitis vinifera* L.) seeds and incorporated in the verum test capsules.

Compound	Amount % (wt∶wt)
**Total catechins**	**25.6**
(+)-catechin	10.9
(−)-epicatechin	12.2
(−)-epicatechin-3-O-gallate	2.5
**Total dimers**	**27.5**
proanthocyanidin B1	7.7
proanthocyanidin B2	8.3
proanthocyanidin B3	2.8
proanthocyanidin B4	1.6
proanthocyanidin B2-gallate	7.1
**Total tri-, tetra- and pentameric proanthocyanidins**	**46.9**

The outcome parameters were measured prior to the start of the supplementation (baseline), and after 4 and 8 weeks of supplementation in the morning after an overnight fast and refraining from smoking and drinking alcohol- and/or caffeine-containing drinks for at least 12 h in advance.

The vascular function measurements took place in a quiet, air-conditioned room of the Academic Hospital Maastricht with a constant temperature of 23°C.

After arrival, subjects rested in supine position for at least 15 min before the measurements commenced. The sequence of the vascular function measurements was for each subject at random, but remained the same for every individual on the three test sessions.

Subsequent to the vascular measurements venous blood was collected. Plasma and serum were obtained by centrifugation at 800 *g* and 4°C for 10 min and immediately processed as described for the individual parameter. Plasma samples for the quantification of the trolox equivalent antioxidant capacity (TEAC), 8-isoprostaglandine F_2α_ (8-iso-PGF_2α_), nitrate and nitrite (NO_x_) and endothelin-1 (ET-1) concentrations were stored at −80°C until analysis.

### Sample size

A conclusive sample size calculation was infeasible for the effects of an 8 weeks MOF supplementation on our primary study parameter, i.e. vascular function assessed as either brachial artery FMD or LDF due to a lack of effect magnitude. In persons at low risk of coronary heart disease, an increase in FMD of 1.4% lowered their Framingham risk by 1% [26]. Assuming a variance of 1.8% for the change in brachial FMD, we would be able to detect a change of 1.4% in FMD in a group of n = [2σ (z_α_+z_β_)^2^](μ_x_−μ_y_)^2^ = 15 subjects with a power of 80% (β = 0.20; z_β_ = 0.84) and an α-value of 0.05 (z_α_ = 1.96) upon the MOF supplementation.

### Measurement of macrovascular function

Macrovascular function was assessed as flow-mediated dilation (FMD) of the brachial artery in accordance with the recommendations of the International Brachial Artery Reactivity Task Force [27]. Details can be found in Text S1. FMD values were calculated as the maximal increase in diameter relative to the baseline diameter (in percentage).

### Measurement of microvascular function

Microvascular function was assessed by measuring skin blood flow responses by means of Laser-Doppler-flowmetry (LDF) following iontophoretical application of 9 subsequent dosages of either acetylcholine (ACh), a mix of ACh and L-N_G_-monomethyl-arginine (L-NMMA) or sodium nitroprusside (SNP) using the Periflux System 5000 (Perimed AB, Stockholm, Sweden). Iontophoretic conditions are given in Text S1. Maximal blood flow perfusion (in percentage of baseline blood flow) and dosage interval resulting in half maximal blood flow response (ED_50_) were determined from the accumulative blood flow response curves by visual inspection.

### Measurement of biochemical vascular parameters

Plasma NO_x_ concentrations were determined by the Griess method as described by Giustarini et al. [28].

Plasma ET-1 was measured by a commercially available radioimmunoassay kit (S2024, Bachem, Switzerland) after plasma extraction by passage through SepPak C18 cartridges (Waters, Netherlands).

Arginase activity in erythrocytes was measured following a modified Schimke's method as described by Corraliza and colleagues [29].

### Measurement of serum lipid levels

Total cholesterol (tChol), low-density lipoprotein cholesterol (LDL), high density lipoprotein cholesterol (HDL) and triglycerides (TG) were quantified by means of enzymatic colorimetric assays on a Roche/Hitachi Modular analyzer (Roche Diagnostics GmbH, Mannheim, Germany).

### Measurement of platelet function

Platelet function was measured in platelet-rich plasma by classical light transmission aggregometry, using a Chronolog aggregometer (Chrono-log Corporation, Havertown, PA, USA; Text S1). Collagen (1.5 mg/L) induced percentage aggregation (CPA) and rate (CPAR) as well as adenosine diphosphate (ADP, 10 µM) induced percentage aggregation (APA) and rate (APAR) were measured in triplicate per subject and time point in the study.

### Measurement of plasma fibrinogen

Fibrinogen (Fib) plasma concentrations were determined on a STA-R Evolution analyzer (Roche Diagnostics GmbH, Mannheim, Germany).

### Measurement of systemic inflammatory parameters

The inflammatory resistance of subjects' blood was investigated *ex vivo* as described by Swennen et al. [30], with some minor modification (Text S1). Tumor necrosis factor (TNF)-α and interleukin (IL)-10 were quantified by means of commercially available ELISA kits (PeliKine Compact human ELISA kits, CLB/Sanquin). The limits of sensitivity were 1 pg/mL for both cytokines.

CRP serum concentrations were measured by particle-enhanced immunoturbidimetry on a Roche/Hitachi Modular analyzer (Roche Diagnostics GmbH, Mannheim, Germany).

### Measurement of redox state parameters

Plasma antioxidant capacity was quantified as TEAC according to Fischer et al [31] and corrected for plasma uric acid concentrations.

Glutathione and glutathione disulphide (GSH/GSSG) concentrations in erythrocytes were assessed as described by Julicher et al. [32] and Griffith [33].

Plasma total 8-iso-PGF_2α_ concentrations were determined after alkaline hydrolysis and solid phase extraction by using a commercially available enzyme immunoassay (Cayman Chemical Company, Ann Arbor, MI, USA).

### Real-time (RT)-Polymerase chain reaction (PCR)

Expression of genes coding for inflammatory mediators and redox enzymes in whole blood were measured by real-time RT-PCR. Details of the procedures as well as the sequences of the primers used are available in Text S1.

### Calculation of the vascular health index (VHI)

As an integrative measure of the diverse effects of the MOF supplementation on vascular health, the VHI was established. This index was calculated per subject after 4 and 8 weeks intervention by adding up the percentage change from baseline of those parameters for which it was expected that an increase indicates a beneficial effect on cardiovascular health. The percentage change from baseline of parameters for which it was expected that a decrease reflects a beneficial health effect were subtracted. The gene expression data were not taken into account, because up- or down regulation of the assessed genes could not be unambiguously related to health benefits. These considerations led to the construction of the following formula:
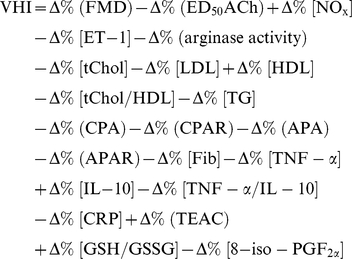



### Statistical methods

All data were tested for normal distribution by visual inspection of the histograms, taking into account the outcomes of the Kolmogorov-Smirnov- and Shapiro-Wilk- tests. Normally distributed data are presented as mean ± SEM. If log-transformation did not result in a normal distribution, data are given as median and range (tables) or 10^th^ and 90^th^ percentiles (figures).

Changes after 4 and 8 weeks intervention with respect to baseline were appraised within a test group by one-tailed paired-samples t-tests in case of normally distributed data and by Wilcoxon Signed Ranks tests in case of not normally distributed data.

Differences between the test groups at each of the 3 time points (baseline, 4 and 8 weeks) as well as in the changes after 4 and 8 weeks with respect to baseline were tested by two-tailed independent samples t-tests in case of normally distributed data and Mann-Whitney U-tests in case of not normally distributed data. Level of significance was set at *P*≤0.05. Statistical analyses were performed using PASW statistics 17.0 (SPSS Inc, Chicago, IL, USA) and GraphPad Prism® (Graphpad Software, Inc., San Diego, CA, USA).

## Results

Both test groups did not differ significantly at baseline with respect to their anthropometric characteristics, smoking habits, blood pressure and serum lipid concentrations (Table 2). Blood pressure values were within the normal range and did not fluctuate significantly over the intervention period. Eleven subjects of the MOF group and 10 subjects of the placebo group exhibited elevated tChol concentrations, i.e. >5 mmol/L [34]. In both groups 9 subjects revealed LDL concentrations >3.2 mmol/L. TG serum concentrations were within the normal range for both groups [35].

**Table 2:** Clinical characteristics of the study population^1^.

	Time (weeks)	MOF (n = 15)	Placebo (n = 13)	*P* value^2^
Age, y	0	46 (30–58)	48 (30–60)	0.982
BMI, kg/m^2^	0	24±1	25±1	0.221
Years of smoking	0	28 (14–45)	29 (15–45)	0.982
Cigarettes/day	0	17 (10–28)	15 (9–30)	0.799
Pack years	0	19 (7–62)	18 (9–54)	0.928
SBP, mmHg	0	114 (103–124)	115 (102–149)	0.580
	4	115 (101–136)	114 (100–154)	0.771
	8	115 (100–134)	108 (100–150)	0.240
DBP, mmHg	0	74 (60–85)	76 (59–99)	0.695
	4	71 (61–85)	74 (58–100)	0.942
	8	72 (56–84)	73 (58–99)	0.645
tChol, mmol/L	0	5.7 (4.0–7.1)	5.7 (3.6–8.5)	0.945
	4	5.7 (4.0–6.3)	5.5 (4.5–8.8)	0.730
	8	5.9 (3.8–6.8)	5.7 (3.6–8.8)	0.800
LDL, mmol/L	0	3.8±0.2	3.9±0.3	0.890
	4	3.7±0.2	3.8±0.3	0.601
	8	3.8±0.2	3.8±0.3	0.821
HDL, mmol/L	0	1.2±0.1	1.3±0.1	0.418
	4	1.2±0.1	1.4±0.1	0.305
	8	1.2±0.1	1.4±0.1	0.190
tChol/HDL ratio	0	4.8 (2.9–8.8)	4.0 (2.9–9.5)	0.433
	4	4.9 (2.6–6.1)	4.0 (2.7–8.5)	0.344
	8	4.9 (2.9–7.5)	4.3 (2.8–9.8)	0.299
TG, mmol/L	0	1.2 (0.9–4.0)	1.4 (0.8–4.3)	0.872
	4	1.6 (0.9–3.7)	1.5 (0.8–3.6)	0.549
	8	1.6 (0.6–2.8)	1.4 (0.7–4.5)	0.533
CRP, mg/L	0	2 (0–6)	1 (0–5)	0.196
	4	2 (0–10)	1 (0–6)	0.317
	8	2 (0–6)	1 (0–6)	0.055
Fibrinogen, g/L	0	3.5 (2.4–4.8)	3.3 (2.8–5.3)	0.460
	4	3.3 (2.4–6.3)	3.5 (2.2–4.9)	0.800
	8	3.9 (2.6–5.7)	3.3 (2.7–4.6)	0.146

1 Values are mean ± SEM or median (range); BMI, body mass index; CRP, C-reactive protein; DBP, diastolic blood pressure; HDL, high density lipoprotein; LDL, low density lipoprotein; MOF, monomeric and oligomeric flavanols; SBP, systolic blood pressure; tChol, total cholesterol; TG, triglycerides.

2 *P* value for between-groups differences assessed by independent Student's t-test or Mann-Whitney U-test where median (range) is indicated.

### Effects of the MOF supplementation on vascular function

Macrovascular function assessed as FMD of the brachial artery did not differ between the MOF and the placebo group throughout the study period (Table 4).

In addition, MOF supplementation did not affect the maximal ACh-induced blood flow response and the maximal endothelial nitric oxide synthase (eNOS)-mediated blood flow response, which was obtained from the maximal blood flow induced by ACh with and without the eNOS inhibitor L-NMMA (data not shown). Large within-subject fluctuations of the blood flow responses upon SNP application reduced the sensitivity of the assessment of these data.

After 4 weeks the ED_50_ of ACh was one dosage interval lower in the MOF group than in the placebo group (*P*<0.05) indicating an increased sensitivity towards the endothelium-dependent vasodilator ACh. However, at the end of the study the ED_50_ in both test groups were not significantly different (*P* = 0.26) (Table 4).

### Effects of the MOF supplementation on biochemical vascular parameters

No effects of the MOF supplementation on plasma nitrite, nitrate and ET-1 concentrations as well as arginase activity in erythrocytes were detected (Table 4).

### Effects of the MOF supplementation on serum lipid concentrations

**Figure 1:**
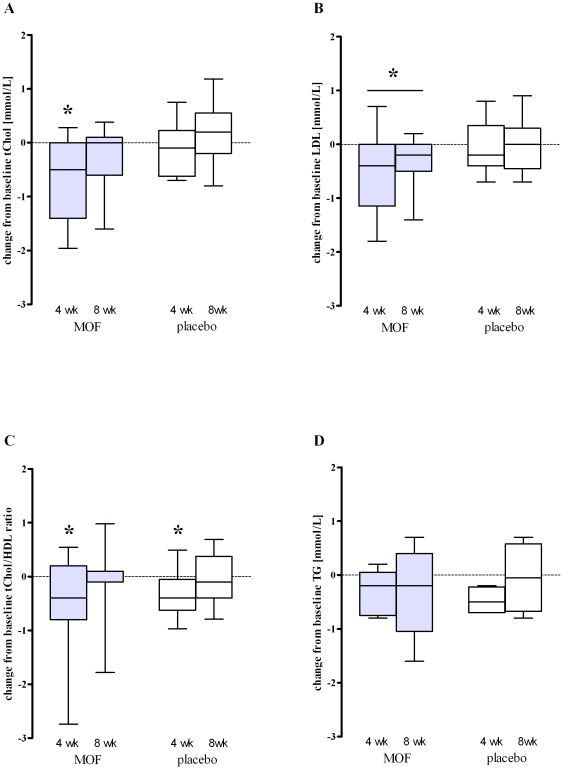
Changes from baseline in median (10^th^ to 90^th^ percentile) serum lipid concentrations after 4 and 8 wk supplementation with either 200 mg/d monomeric and oligomeric flavanols (MOF) or placebo: (A) total cholesterol (tChol) concentrations of subjects with tChol baseline concentrations >5.0 mmol/L (MOF group: n = 11, placebo group: n = 10); (B) low density lipoprotein (LDL) concentrations of subjects with LDL baseline concentrations >3.2 mmol/L (MOF group: n = 9, placebo group: n = 9); (C) ratio of tChol to high density lipoprotein (HDL) of subjects with tChol baseline concentrations >5.0 mmol/L (MOF group: n = 11, placebo group: n = 10); (D) triglycerides (TG) concentrations of subjects with TG baseline concentrations >1.7 mmol/L (MOF group: n = 5, placebo group: n = 4). Within-group changes were appraised by Wilcoxon Signed Ranks test, between-group changes by Mann-Whitney U test; ^*^Significantly different from baseline in the same group, *P*<0.05. There were no significant differences between the MOF and the placebo group at the same time points.

Serum lipid concentrations did not change significantly during the 8 weeks intervention in both test groups (Table 2). However, in the subgroup of individuals with baseline tChol>5 mmol/L (n = 11) MOF supplementation significantly lowered tChol by 9% after 4 weeks (*P*<0.05 vs. baseline) and by 5% after 8 weeks (*P* = 0.05 vs. baseline). In contrast, in the similar placebo subgroup (n = 10), no significant effects on tChol were seen during the study (Figure 1A).

LDL concentrations in the subgroup with baseline levels >3.2 mmol/L (n = 9) decreased significantly by 11% after 4 weeks (*P*<0.05 vs. baseline) and 7% after 8 weeks MOF intake (*P*<0.05 vs. baseline; Figure 1B). In relation to that, in the similar placebo subgroup (n = 9) no significant effect on LDL was observed.

In the MOF and the placebo subgroup the tChol-HDL-ratio did not differ significantly during the intervention (Figure 1C).

5 subjects of the MOF group and 4 of the placebo group had TG concentrations >1.7 mmol/L, which is defined by the American Heart Association as “borderline high” [35]. After 4 weeks the average TG concentrations dropped by 13% (MOF, *P* = 0.16 vs. baseline) and 16% (placebo, *P* = 0.06 vs. baseline). While in the placebo subgroup TG concentrations returned to baseline after 8 weeks (*P* = 0.44 vs. baseline), in the MOF subgroup TG concentrations remained on the low 4 weeks level (*P* = 0.31 vs. baseline; Figure 1D).

### Effects of the MOF supplementation on platelet aggregation *ex vivo* and plasma fibrinogen levels

Whereas the total percentage of collagen-and ADP-induced platelet aggregation did not change by the 8 weeks MOF supplementation (Table 3), the rate of the collagen-induced aggregation was lower in the MOF group than in the placebo group after 8 weeks (*P*<0.01, Table 3). In contrast to this, the rate of the ADP-induced aggregation increased significantly from baseline in the MOF group (*P*<0.05).

**Table 3:** Platelet aggregation parameters.^1^

	Time (weeks)	MOF (n = 15)	Placebo (n = 13)	*P* value^2^
APA, %	0	75 (65–86)	77 (58–96)	0.872
	4	74 (65–81)	71 (48–84)	0.117
	8	74 (57–85)	74 (62–83)	0.835
APAR, slope	0	102 (59–135)	109 (74–133)	0.140
	4	107 (62–127)	97 (83–141)	0.872
	8	112 (77–139)	101 (81–138)	0.278
CPA, %	0	76 (70–81)	77 (71–96)	0.151
	4	78 (65–84)	78 (71–83)	0.746
	8	78 (60–81)	79 (71–82)	0.782
CPAR, slope	0	88±13	94±17	0.338
	4	92±12	95±11	0.486
	8	86±12	99±8	0.003

1 Values are mean ± SEM or median (range); APA, ADP-induced platelet aggregation; APAR, ADP-induced platelet aggregation rate; CPA, collagen-induced platelet aggregation; CPAR, collagen-induced platelet aggregation rate.

2 *P* value for between-groups differences assessed by independent Student's t-test or Mann-Whitney U-test where median (range) is indicated.

**Table 4:** Macro- and microvascular function and biochemical vascular parameters.^1^

	Time (weeks)	MOF (n = 15)	Placebo (n = 13)	*P* value^2^
FMD, %	0	3.5 (0.9–5.8)	4.8 (0–11.8)	0.294
	4	3.0 (−0.8–11.5)	4.7 (1.3–10.7)	0.149
	8	3.5 (0.8–5.6)	3.7 (1.9–5.2)	0.678
ED_50_ACh	0	5 (2–7)	5 (0–7)	0.384
	4	4 (3–6)	5 (0–8)	0.039
	8	4 (2–6)	3 (0–6)	0.257
NO_x_,µM	0	25.8±2.1	24.3±3.3	0.518
	4	23.8±1.7	25.7±2.9	0.637
	8	23.2±1.8	21.9±2.2	0.525
ET-1, pmol/L	0	6.6 (4.8–7.9)	6.8 (5.7–8.4)	0.322
	4	6.2 (5.0–12.0)	6.3 (4.9–8.4)	0.790
	8	6.3 (4.9–7.3)	6.3 (5.6–7.8)	0.596
Arg. activity, µM urea×mg protein^−1^×h^−1^	0	1.6 (1.2–3.4)	1.6 (1.0–2.0)	0.627
	4	1.7 (1.1–2.9)	1.4 (1.0–2.0)	0.277
	8	1.7 (1.2–3.0)	1.5 (1.1–2.0)	0.391

1 Values are mean ± SEM or median (range); Arg. activity, erythrocytes' arginase activity; ED_50_ACh, ACh dose interval resulting in 50% of the maximum dermal blood flow response; ET-1, plasma endothelin-1; FMD, flow mediated dilation; MOF, monomeric and oligomeric flavanols; NO_x_, plasma nitrite and nitrate.

2 *P* value for between-groups differences assessed by independent Student's t-test or Mann-Whitney U-test where median (range) is indicated.

Plasma fibrinogen concentrations were unaffected by the MOF supplementation (Table 2).

### Effects of the MOF supplementation on systemic inflammatory parameters

**Figure 2:**
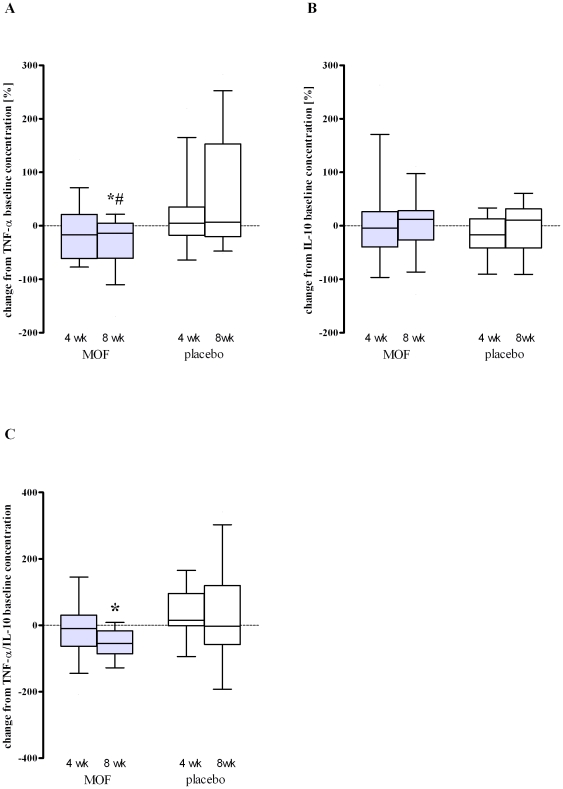
Changes from baseline in median (10^th^ to 90^th^ percentile) cytokine concentrations released upon LPS-addition (100 ng/mL) to subjects' blood *ex vivo* after 4 and 8 wk supplementation with either 200 mg/d monomeric and oligomeric flavanols (MOF, n = 15) or placebo (n = 13): (A) TNF-α, percentage change; (B) IL-10, percentage change; (C) ratio of TNF-α to IL-10, absolute change. Within-group changes were appraised by Wilcoxon Signed Ranks test, between-group changes by Mann-Whitney U test; ^*^Significantly different from baseline in the same group, *P*<0.05. ^#^Significant difference between groups at the same time, *P*<0.05.

*Ex vivo* LPS-induced release of the proinflammatory cytokine TNF-α decreased from baseline by 14% (*P*<0.05) after 8 weeks MOF supplementation. This reduction was also significant compared to the placebo group at 8 weeks (*P*<0.05; Figure 2A). The release of the anti-inflammatory IL-10 did not deviate significantly from baseline in the MOF supplemented group and was similar to the alterations observed in the placebo group. However, the significantly lower ratio of the pro- and the anti-inflammatory cytokine (TNF-α/IL-10: baseline median = 105 vs. 8 weeks median = 62, *P*<0.05) underlines the anti-inflammatory effect of the MOF supplementation.

Moreover, the MOF supplementation significantly attenuated gene expression of the cytokines IL-6 (after 4 weeks: −18%, *P*<0.05 vs. baseline) as well as TNF-α (after 8 weeks: −12%, *P*<0.05 vs. baseline) and IL-10 (after 8 weeks: −27%, *P*<0.05 vs. baseline) in whole blood (Figure S2A, C, E). Contrary, MOF supplementation did not alter serum CRP concentrations (Table 2) and the expression of genes coding IL-1β and IL-8, NOS2, nuclear factor of kappa light polypeptide gene enhancer in B-cells inhibitor alpha, intercellular adhesion molecule 1 and vascular cell adhesion molecule 1 (Figure S2B, D, F, G, H, I).

### Effects of the MOF supplementation on redox state parameters and oxidative stress

**Figure 3:**
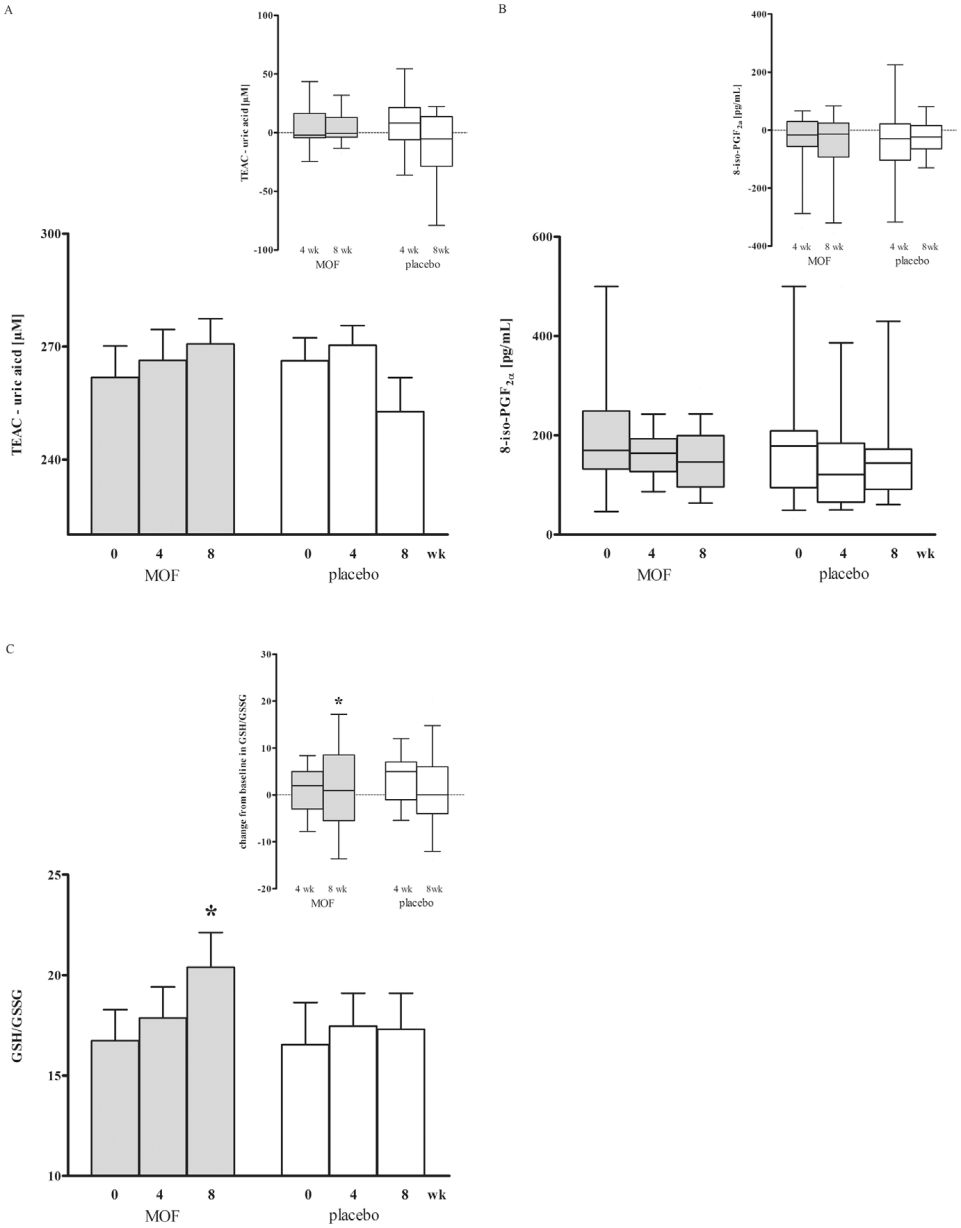
Mean ± SEM (bars) or median (10^th^ and 90^th^ percentile) (box and whiskers) antioxidant capacity of plasma measured as trolox equivalent antioxidant capacity (TEAC) and corrected for uric acid plasma concentrations (A), ratio of glutathione (GSH) to glutathione disulphide (GSSG) in erythrocytes (B) and 8-isoprostaglandine F_2α_ (8-iso-PGF_2α_) plasma concentrations of subjects at baseline (0 wk) and after 4 and 8 wk supplementation with either 200 mg/d monomeric and oligomeric flavanols (MOF, n = 15) or placebo (n = 13). The insert displays for each of the parameter the changes from baseline after 4 and 8 weeks intervention. Within-group changes were appraised by either one-tailed paired-samples t-test (bar plots) or Wilcoxon Signed Ranks test (box and whiskers plots), between-group changes by either two-tailed independent samples t-test (bar plots) or Mann-Whitney U test (box and whiskers plots); ^*^Significantly different from baseline in the same group, *P*<0.05. There were no significant differences between the MOF and the placebo group at the same time points.

The antioxidant capacity of plasma, quantified as TEAC and corrected for uric acid as major antioxidant in blood, tended to increase by the 8 weeks MOF supplementation. However, significant differences compared to the placebo group could not be found (Figure 3A).

Circulating levels of the lipid peroxidation product 8-iso-PGF_2α_ were not affected by the 8 weeks supplementation with MOF (Figure 3B).

Subjects allocated to the MOF group revealed 22% higher GSH/GSSG concentrations in erythrocytes after 8 weeks compared to baseline (*P*<0.05, Figure 3C). This increase appeared to originate from a reduction in the GSSG quantities rather than from a substantial increase in the amount of erythrocytes' GSH (data not shown).

No effect on the systemic expression of genes coding catalase (CAT), glutathione peroxidase (GPX)1 and 4, glutathione reductase (GSR), heme oxygenase 1 (HMOX1) and superoxide dismutase 2 (SOD2) were observed in both groups (Figures S3A–F). In the MOF group the expression of CAT, GSR and HMOX1 tended to decline after 8 weeks regarding baseline (Figures S3A, 5D, 5E).

### Effects of the MOF supplementation on the VHI

**Figure 4:**
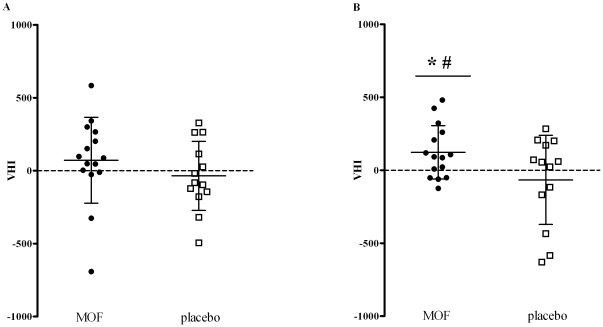
Mean ± SD vascular health index (VHI) of individual subjects after 4 (A) and 8 wk (B) supplementation with either 200 mg/d monomeric and oligomeric flavanols (MOF, n = 15) or placebo (n = 13). Within-group changes were appraised by one-tailed paired-samples t-test, between-group changes by two-tailed independent samples t-test; ^*^Significantly different from baseline in the same group, *P*<0.05. ^#^Significant difference between groups at the same time, *P*<0.05.

The VHI is used to integrate the multiple effects monitored in the present study. After 4 weeks the VHI tended to be higher in the MOF group (mean ± SD = 62±75) than in the placebo group (mean ± SD = −35±61, Figure 4A). The difference increased after 8 weeks resulting in significantly higher VHI levels in the MOF group (mean ± SD = 123±47, *P*<0.05 vs. baseline) compared to the placebo group (mean ± SD = −66±79, *P*≤0.05, Figure 4B).

## Discussion

The beneficial effects of food derived flavanols on vascular health are becoming increasingly aware. However, the clinical efficacy of these compounds in particular long-term is yet unclear and limits their purposive application in daily medical practice. The results of the present pilot study unveiled the potency of MOF from *Vitis vinifera* L. to improve vascular health when regularly applied for 8 weeks in relatively small amounts of 200 mg per day in addition to the normal diet. The frequently observed positive trends on single endpoints add up to a distinctive overall vascular health benefit as indicated by a significantly higher vascular health index compared to placebo.

In order to study health effects of dietary supplements the study population, while generally healthy, should have or produce under stress elevated levels of the relevant parameters. The disturbances should be mild so that no pharmacological intervention is required. The group of 28 smokers recruited in the present trial fulfilled these criteria adequately. The majority of the volunteers exhibited a number of established cardiovascular risk factors, e.g. elevated serum total cholesterol and LDL [34] as well as slightly increased CRP concentrations [34], [36], but were neither diagnosed with a vascular disease nor on cardiovascular medication. Notably, the averaged endothelial function of smokers measured non-invasively by means of brachial artery FMD at baseline (mean FMD ± SD = 4±3%) was in good agreement with data from Celermajer and colleagues who reported impaired macrovascular function of smokers (mean FMD ± SD = 4±4%) compared to age- and sex-matched non-smokers (mean FMD ± SD = 10±3%) [37]. Considering cardiovascular risk classification by the Framingham Risk Scores [38] and by an approach based on brachial artery FMD values [26], indicates that the healthy subjects in our test groups might be at a moderate cardiovascular risk.

Interestingly, it has been found that in a population at low cardiovascular risk FMD values are inversely correlated with cardiovascular risk, while in populations at intermediate or high risk this correlation diminishes and disappears, respectively [26]. This phenomenon has been explained by the observation that people with elevated cardiovascular risk possess a limited distensibility of the brachial artery [39]. As a result FMD values may not reflect any longer an exclusively endothelial NO^•^-mediated response. Irrespective from this aspect, evidence is lacking yet that MOF may improve macrovascular function in humans upon a long-term ingestion period, i.e. more than 4 weeks [40]. Interestingly, dermal microvasculature became transiently more sensitive to the endothelium-dependent stimulus ACh upon 4 weeks MOF intake. However, we cannot rule out that this effect was due to chance, since after 8 weeks of intervention a similar increase in sensitivity was observed in the placebo group.

Despite a wealth of animal studies demonstrating serum cholesterol lowering effects of various mixtures of grape seed flavanols, studies in humans are less conclusive [22], [41]. Species differences as well as variations in flavanolic composition might result in altered clinical efficacy [42]. Our trial clearly emphasized the potential of MOF to reduce hypercholesterolemia to a significant and clinically relevant extent over an 8 weeks consumption period. Although the cholesterol-lowering effects were more pronounced after 4 than after 8 weeks, the median LDL concentrations remained 0.2 mmol/L lower at the end of the study compared to baseline. The same efficacy was reported for green tea and soy protein isolate [41] and was estimated to reduce all-cause mortality by 3% and CHD-related mortality and total CHD events by 6% [43].

Moreover, our study showed a significant enhancement of the smokers' resistance against inflammatory stimuli, such as bacterial endotoxin, upon the 8 weeks MOF supplementation. Remarkably, MOF alleviated the inflammatory response in blood not only by mitigating the LPS-induced secretion of the pro-inflammatory cytokine TNF-α, but also by enhancing the release of the anti-inflammatory cytokine IL-10. Likewise we could demonstrate that the MOF-mediated increase in systemic inflammatory resistance extends in humans to the modulation of the gene expression of inflammatory cytokines, in particular TNF-α, IL-6 and IL-10. Although cell culture experiments suggest a direct interference of isolated MOF with NF-κB-mediated gene expression [44], the unaltered expression of the inhibitory subunit NFKBIA did not indicate an effect on the NF-κB pathway by MOF. Additionally, we could not detect significant changes in the gene expression of adhesion molecules, which are also regulated by the transcription factor NF-κB.

Smokers are known to exhibit elevated levels of oxidative stress, which has been established among others as reduced antioxidant status [45], [46] as well as a rise in biomarkers for lipid peroxidation such as F2 isoprostanes [11], [47]. We thus determined the systemic extra- and intracellular redox state of the smokers under MOF supplementation by measuring TEAC in plasma, GSH/GSSG concentrations in erythrocytes and gene expression levels of the redox enzymes CAT, GPX1, GPX4, GSR, HMOX1 and SOD2 in blood. The trend of increasing plasma TEAC values and the considerable augmentation of the GSH/GSSG ratio in erythrocytes indicate that MOF improve extra- and intracellular redox status in the circulation. These effects, however, could not be ascribed to alterations of gene expression of the most important enzymatic antioxidant defense systems in circulatory white blood cells as discussed above. The systemic oxidative stress levels assessed as plasma concentrations of the lipid peroxidation marker 8-iso-PGF_2α_ were unaffected upon the 8 weeks MOF intake. Similarly, it has been reported that a daily ingestion of 1000 mg grape seed polyphenols for 6 weeks did not lower plasma isoprostane levels in hypertensive subjects.

Our study also revealed that chronic MOF consumption did not affect ADP- and collagen stimulated platelet aggregation *ex vivo*. Whereas platelet-inhibiting effects could be clearly proven in humans upon ingestion of cocoa-related products [48], the clinical evidence for MOF from *Vitis vinifera* L. is less clear and might be related to a difference in polyphenolic composition and dose [48]. Moreover, experiments with isolated human platelets suggest that grape seed extract inhibits aggregation only in quite high concentrations (100 mg/L blood) [49], which fairly exceed plasma levels achievable in vivo. A prothrombotic state has also been associated with elevated fibrinogen plasma concentrations, typically occurring in smokers [50]. This glycoprotein has been identified to promote plasma viscosity, platelet aggregation, coagulation and inflammation, thereby fueling atherogenesis. Therefore, agents that lower circulatory fibrinogen might be valuable [51]. The 8 week MOF intervention, however, remained ineffective in this respect.

In the past decade *in vitro* and *in vivo* studies unraveled several molecular mechanisms of flavanols that are essential in the beneficial mediation of their vascular effects. These mechanisms comprise a reduction in arginase activity that may contribute among others to an increased endothelial NO^•^ level [52], as well as a decline in plasma ET-1 concentration [53]. In the setting of our clinical trial we could not find significant changes in plasma nitrate and nitrite concentration measured as surrogate for endothelial NO^•^ production [54], as well as in arginase activity in erythrocytes and ET-1 plasma concentration.

Already during the screening of the volunteers we carefully checked by means of an investigator-guided interview and a suitable questionnaire the intake of coffee, tea and chocolate containing drinks as well as the consumption of chocolate and chocolate containing food products and alcoholic beverages, including beer, red and white wine. Also the use of any vitamins and/or food supplements, adherence to a vegetarian and vegan diet, a regular (at least 3 times a day) eating pattern as well as fluctuations in body weight were evaluated and had to comply with the in- and exclusion criteria of the study (see Methods, “Subjects and study design”). In this way we could exclude beforehand extreme and unusual behaviors and recruit a quite homogenous group of typical Dutch middle-aged men [55]. Since, however, we neither used food frequency questionnaires to determine the exact daily food intake before and during the study nor measured plasma levels of relevant flavanol metabolites, we could not accurately assess the total intake of theses compounds in both test groups. The rational for imposing no restriction regarding diet and lifestyle was, that in this way clinical data were collected on the vascular health effects of a relatively low-dose supplementation with MOF under real-life conditions.

Food scientists are facing the challenge to proof the clinical efficacy of nutrients that modulate human physiology in a subtle and non-specific manner [25]. In drug research, where the one-target-one-hit concept is imperative, randomized clinical trials with a well-defined single endpoint are the gold standard of efficacy testing. However, a single endpoint neither sufficiently reflects the multifarious nature of nutrients' functions in humans nor the complexity of pathomechanisms underlying virtually all diseases.

Our study pioneered the implementation of a pragmatic solution for this problem: Carefully select a representative panel of markers that reflect the major relevant pathological aberrations and integrate all measured effects into a global health index. This concept is in particular elegant, since it can easily be applied to practically any other field of diseases.

In conclusion, our integrative multi-biomarker approach proved to be a sensitive and therefore powerful strategy to unveil the pleiotropic benefit of an 8 weeks supplementation with 200 mg/d MOF on vascular health in humans.

## Supporting Information

Figures S1 **Flow of participants through the study.**
(DOC)

Figure S2 **Expression of genes encoding inflammatory mediators and adhesion molecules.**
(EPS)

Figure S3 **Expression of genes encoding redox enzymes.**
(EPS)

Text S1 **Additional information regarding the methods applied.**
(DOC)

Protocol S1 **Study protocol.**
(DOC)

Checklist S1 **CONSORT 2010 checklist of information to include when reporting a randomized trial.**
(DOC)
