# Supplementary material for: Pleiotropic Benefit of Monomeric and Oligomeric Flavanols on Vascular Health - A Randomized Controlled Clinical Pilot Study
Source: PLoS One. 2011 Dec 8;6(12):e28460. doi: 10.1371/journal.pone.0028460 (PMC3234272; doi:10.1371/journal.pone.0028460)
Supplement: Figures S1 — Flow of participants through the study. (DOC) [file pone.0028460.s001.doc]

**Figure S1**

CONSORT 2010 Flow Diagram

**Allocation**

**Analysis**

**Follow-Up**

**Enrollment**

Assessed for eligibility (n=85)

Excluded (n=52)

♦  Not meeting inclusion criteria (n=15)

♦  Declined to participate (n=6)

♦  Other reasons (n=31)

Analysed (n=15)
♦ Excluded from analysis (n=0)

Lost to follow-up (n=0)

Discontinued intervention (n=0)

Allocated to MOF intervention (n=16)

♦ Received allocated intervention (n=15)

♦ Did not receive allocated intervention (did not show up on 1^st^ study day) (n=1)

Lost to follow-up (personal reasons) (n=1)

Discontinued intervention (n=0)

Allocated to placebo intervention (n=17)

♦ Received allocated intervention (n=14)

♦ Did not receive allocated intervention (1 subject did not show up on 1^st^ study day, 1 subjects became ill before 1^st^ study day, 1 subject was excluded due to seriously elevated blood pressure on 1^st^ study day) (n=3)

Analysed (n=13)
♦ Excluded from analysis (n=0)

Randomized (n=33)
