# Supplementary material for: Pleiotropic Benefit of Monomeric and Oligomeric Flavanols on Vascular Health - A Randomized Controlled Clinical Pilot Study
Source: PLoS One. 2011 Dec 8;6(12):e28460. doi: 10.1371/journal.pone.0028460 (PMC3234272; doi:10.1371/journal.pone.0028460)
Supplement: Text S1 — Additional information regarding the methods applied. (DOC) [file pone.0028460.s004.doc]

**Methods**

**Measurement of macrovascular function**

Macrovascular function was assessed as flow-mediated dilation (FMD) of the brachial artery in accordance with the International Brachial Artery Reactivity Task Force [1]. In brief, the brachial artery was imaged longitudinally 2-10 cm above the antecubital fossa in brightness mode (B-mode) by a clinical ultrasound scanner (7.5 MHz vascular probe, Picus I, Esaote Biomedica, Genoa, Italy). FMD measurements were made in combined echo Doppler/B-mode, with a single lead ECG tracing displayed as beat-to-beat reference. The size of the Doppler sampling volume was less than half the vessel diameter and positioned in the center of the vessel. During the FMD measurement, the dual mode ultrasound images were recorded simultaneously and continuously on DVD over the entire measurement duration (13 min.). A rapid inflation/deflation pneumatic cuff was positioned around the forearm. After recording baseline diameter and blood flow for 3 minutes at rest, the forearm cuff was inflated to a pressure of 50 mmHg above systolic blood pressure to block forearm blood flow, creating peripherally a hypoxic state. After 5 minutes of occlusion the cuff was deflated, inducing a high-flow (hyperemic) state. The associated temporary increase in shear stress causes a transient dilation of the brachial artery, which was recorded for another 5 minutes. All FMD measurements were carried out by one and the same well-trained and experienced investigator.

The Doppler/B-mode video images were analyzed using a semi-automated image-analysis algorithm developed in-house (Dept. of Biomedical Engineering, Maastricht University, The Netherlands), providing beat-to-beat estimates of end-diastolic diameter and blood flow velocity averaged over the cardiac cycle. Both blood flow velocity and diameter curves were smoothed with a 17-point median filter. The FMD response was calculated as the ratio of the maximum hyperemic change in diameter and baseline diameter, expressed as a percentage of the baseline diameter.

At least 4 times before and during the FMD measurements blood pressure and heart rate were monitored in 3 min. intervals by an automated sphygmomanometer (Dinamap xT, Critikon Corporation, Tampa, FL, USA) on the contralateral arm. Average blood pressure and heart rate were calculated based on measurements 2, 3 and 4 (first readings discarded).

**Measurement of microvascular function**

The cutaneous blood flow response upon the iontophoretical application of vasodilatory compounds was assessed by Laser-Doppler flowmetry (LDF) on the dorsal surface of the intermediate phalanges. Subjects were instructed not to use any skin moisturizing crèmes or lotions during the last three days before a measurement. After application of the drug delivery electrode to its intended position skin temperature could be monitored and kept at a constant level of 32°C throughout the measurements. Acetylcholine (ACh, 1% (m/v) in water) and a combination of ACh (1% (m/v)) and L-N_G_-monomethyl-arginine (L-NMMA, 1% (m/v)) were delivered by anodal iontophoresis in 9 subsequent dosages of 0.1 mA for 20 sec (2 mC) in 1 min intervals. Sodium nitroprusside (SNP, 1% (m/v)) was delivered by cathodal iontophoresis in 9 subsequent dosages of 0.2 mA for 20 sec (4 mC) in 1.5 min intervals. ACh, the mix of ACh and L-NMMA and SNP were delivered on different fingers, either the index, middle or the ring finger. The sequence and location of the measurements was kept constant per volunteer throughout the study. During the entire measurements subjects were asked to quietly lie on the bed and to avoid speaking and moving their limbs. In order to avoid the introduction of inter-investigator variability all measurements were carried out by the same researcher.

The accumulative blood flow response curves were digitally recorded and accessible via the Perimed software PeriSoft for Windows, version 2.50.

**Measurement of platelet function**

Light transmission aggregometry (LTA) was immediately performed after collection of venous blood in tubes containing sodium citrate as anticoagulant. Platelet rich plasma (PRP) was obtained after immediate centrifugation at 240 *g* for 15 min. Platelet poor plasma (PPP) was prepared after centrifugation at 2630 *g* for 10 minutes. PPP was carefully removed from the blood cells and centrifuged again at 2630 *g* for 10 minutes. PRP was adjusted with PPP to 25🞩10^10^ platelets/L by means of a thrombo-counter (Coulter Electronics, Luton, UK). After 2 min. baseline measurement either collagen (final assay concentration 1.5 µg/mL) or adenosine diphosphate (ADP, final concentration 10 µM) was added to the stirred PRP. Platelet aggregation was recorded as an increase in light transmission over 8 min. All centrifugation steps and the platelet aggregation measurements were performed at room temperature.

**Measurement of systemic inflammatory markers**

In order to determine systemic inflammatory resistance freshly collected heparinized blood was aliquoted into 24-well sterile plates and diluted 1:4 with RPMI 1640 (Invitrogen). Cytokine production in the blood was induced by the addition of 100 ng/mL LPS (O26:B6, Sigma). After incubating the plates in 5% CO_2_ at 37°C for 6 h, the cell-free supernatant was collected by centrifugation (19,720 *g*, 10 min, 4°C) and stored at -80°C until quantiﬁcation of the cytokines.

**Reverse-transcriptase-polymerase chain reaction (RT-PCR)**

In order to preserve RNA freshly collected whole blood was added to RNALater® (Ambion, Austin, TX, USA) and stored at -80˚C. Total RNA was isolated from whole blood using RiboPure-Blood kit (Ambion, Austin TX, USA) and A260/280 measured to determine concentration and purity. 500 ng of total RNA was reverse transcribed using iScript cDNA synthesis kit (Bio-Rad, Hercules, CA, USA) followed by DNA digestion with DNase I. Quantitative real-time PCR (qPCR) was performed in a total volume of 25 µl containing 5 µl cDNA, 2.5 µl (3 µM) of each primer and 12.5 µl SensiMix SYBR & Fluorescein Kit (Quantace, London, UK). Primer sequences are listed in table 1 of the online-only Data Supplement. The cycling conditions comprised an initial denaturation step at 95ºC for 10 min. followed by 40 cycles of 15 sec. at 95ºC and 45 sec. at 60ºC using an iCycler (Bio-Rad, Hercules, CA, USA). After completion of the cycling process, a melting curve (65ºC - 95ºC) was produced to confirm product formation and purity. Data were analyzed using the MyIQ software system (Bio-Rad, Hercules CA, USA). Data were expressed as relative gene expression values (RE) by calculating for each time point in the study the ΔCt values under consideration of glyceraldehyde-3-phosphate dehydrogenase (GAPDH) as housekeeping gene and normalizing the values at 4 and 8 weeks for baseline ΔCt values.

**References**

1. Corretti MC, Anderson TJ, Benjamin EJ, Celermajer D, Charbonneau F, et al. (2002) Guidelines for the ultrasound assessment of endothelial-dependent flow-mediated vasodilation of the brachial artery: A report of the International Brachial Artery Reactivity Task Force. J Am Coll Cardiol 39: 257-265.

**­**

**Figure Legend**

**Figure S2.** Mean ± SEM (bars or median (10^th^ and 90^th^ percentile) (box and whiskers) relative gene expression (RE) of tumor necrosis factor alpha (TNF) (A), interleukin 1 beta (IL1B) (B), interleukin 6 (IL6) (C), interleukin 8 (IL8) (D), interleukin 10 (IL10) (E), nitric oxide synthase 2 (NOS2) (F), nuclear factor of kappa light polypeptide gene enhancer in B-cells inhibitor alpha (NFKBIA) (G), intercellular adhesion molecule 1 (ICAM1) (H), vascular adhesion molecule 1 (VCAM 1) (I) in blood of subjects at baseline (0 wk) and after 4 and 8 wk supplementation with either 200 mg/d monomeric and oligomeric flavanols (MOF, n = 15) or placebo (n = 13). Within-group changes were appraised by either one-tailed paired-samples t-test (bar plots) or Wilcoxon Signed Ranks test (box and whiskers plots), between-group changes by either two-tailed independent samples t-test (bar plots) or Mann-Whitney U test (box and whiskers plots); ^*^Significantly different from baseline in the same group, *P* < 0.05. There were no significant differences between the MOF and the placebo group at the same time points.

**Figure S3.** Mean ± SEM (bars) or median (10^th^ and 90^th^ percentile) (box and whiskers) relative gene expression (RE) of catalase (CAT) (A), glutathione peroxidase 1 (GPX1) (B), glutathione peroxidase 4 (GPX4) (C), glutathione reductase (GSR) (D), heme oxygenase 1 (HMOX1) (E), superoxide dismutase 2 (SOD2) (F) in blood of subjects at baseline (0 wk) and after 4 and 8 wk supplementation with either 200 mg/d monomeric and oligomeric flavanols (MOF, n = 15) or placebo (n = 13). Within-group changes were appraised by either one-tailed paired-samples t-test (bar plots) or Wilcoxon Signed Ranks test (box and whiskers plots), between-group changes by either two-tailed independent samples t-test (bar plots) or Mann-Whitney U test (box and whiskers plots). There were no significant differences from baseline in the same group and between both groups at the same time points.

**Table S1.** Primer sequences used for the RT-PCR experiments

| Gene | Primer | Sequence |
| --- | --- | --- |
| CAT^1^ | Forward | GACTGACCAGGGCATCAAAAA |
|  | Reverse | CGGATGCCATAGTCAGGATCTT |
| GAPDH | Forward | GCACCACCAACTGCTTAGCA |
|  | Reverse | TGGCAGTGATGGCATGGA |
| GPX1 | Forward | CCCGTGCAACCAGTTTGG |
|  | Reverse | CGGACGTACTTGAGGGAATTCA |
| GPX4 | Forward | CCTTTGCCGCCTACTGAAG |
|  | Reverse | CACAGCGCCAGTCGTCC |
| GSR | Forward | CAAGCTGGGTGGCACTTG |
|  | Reverse | TTGGAAAGCCATAATCAGCA |
| HMOX1 | Forward | CTTCTTCACCTTCCCCAACA |
|  | Reverse | GCTCTGGTCCTTGGTGTCAT |
| ICAM1 | Forward | CTGAGCAATGTGCAAGAAGATAGC |
|  | Reverse | CCCGTTCTGGAGTCCAGTACA |
| IL1B | Forward | TCCCCAGCCCTTTTGTTGA |
|  | Reverse | TTAGAACCAAATGTGGCCGTG |
| IL6 | Forward | AGTGAGGAACAAGCCAGAGC |
|  | Reverse | GTCAGGGGTGGTTATTGCAT |
| IL8 | Forward | GGACAAGAGCCAGGAAGAAA |
|  | Reverse | AAATTTGGGGTGGAAAGGTT |
| IL10 | Forward | GCTGTCATCGATTTCTTCCC |
|  | Reverse | CTCATGGCTTTGTAGATGCCT |
| NFKBIA | Forward | CTACACCTTGCCTGTGAGCA |
|  | Reverse | TCCTGAGCATTGACATCAGC |
| NOS2 | Forward | TTCAAGACCAAATTCCACCAG |
|  | Reverse | ATTCTGCTGCTTGCTGAGGT |
| SOD2 | Forward | ATCAGGATCCACTGCAAGGAA |
|  | Reverse | CGTGCTCCCACACATCAATC |
| TNF | Forward | TCAATCGGCCCGACTATCTC |
|  | Reverse | CAGGGCAATGATCCCAAAGT |
| VCAM1 | Forward | TGGGAAAAACAGAAAAGAGGTG |
|  | Reverse | GTCTCCAATCTGAGCAGCAA |

^1^CAT, catalase; GAPDH, glyceraldehyde-3-phosphate dehydrogenase; GPX1, glutathione peroxidase 1; GPX4, glutathione peroxidase 4; GSR, glutathione reductase; HMOX1, hemeoxygenase 1; ICAM1, intercellular adhesion molecule 1; IL1B, interleukin 1 beta; NFKBIA, nuclear factor of kappa light polypeptide gene enhancer in B-cells inhibitor alpha; NOS2, nitric oxide synthase 2; SOD2, superoxide dismutase 2; VCAM1, vascular adhesion molecule 1.
