# Supplementary material for: Pleiotropic Benefit of Monomeric and Oligomeric Flavanols on Vascular Health - A Randomized Controlled Clinical Pilot Study
Source: PLoS One. 2011 Dec 8;6(12):e28460. doi: 10.1371/journal.pone.0028460 (PMC3234272; doi:10.1371/journal.pone.0028460)
Supplement: Protocol S1 — Study protocol. (DOC) [file pone.0028460.s005.doc]

**The effects of oligomeric procyanidins (OPCs) on vascular function, biomarkers of oxidative stress and inflammation in smokers: a pilot study**

**Clinical study protocol**

**Doc: INC001**

**Rev: 04**

**Date: July 22, 2009**

**Written by: Antje Weseler, PhD**

| **Protocol ID** | INC 001 |
| --- | --- |
| **Short title** | Cardiovascular effects of OPCs in smokers |
| **Version** | 04 |
| **Date** | 22 July, 2009 |
| **Coordinating investigator/project leader** | Antje R. Weseler, PhD  Dept. of Pharmacology and Toxicology  Maastricht University  Universiteitssingel 50  6229 ER Maastricht  The Netherlands  Tel: +31-(0)43-3882916  Fax: +31-(0)43-3884149 |
| **Principal investigators**  **(in Dutch: hoofdonderzoeker/uitvoerder)** | Antje R. Weseler, PhD  Dept. of Pharmacology and Toxicology  Maastricht University  Universiteitssingel 50  6229 ER Maastricht  The Netherlands  Tel: +31-(0)43-3882916 |
| **Sponsor**  **(in Dutch: verrichter/opdrachtgever)** | Maastricht University  Dept. of Pharmacology & Toxicology  PO Box 616  6200 MD Maastricht  The Netherlands |
| **Responsible medical doctor** | Ger Koek, MD, PhD  Dept. of Internal Medicine  Maastricht University  Universiteitssingel 50  6229 ER Maastricht  The Netherlands  Tel: +31-(0)43-3877028 |

| **Independent physician** | Thomas K. A. Wierema, MD, PhD  Dept. of Internal Medicine  University Hospital Maastricht Cardiovascular Research Institute Maastricht PO Box 5800 6202 AZ Maastricht The Netherlands Tel: +31-(0)43-3877005 |
| --- | --- |
| **Laboratory sites** | Dept. of Pharmacology and Toxicology  Maastricht University  Universiteitssingel 50  6229 ER Maastricht  The Netherlands  Tel: +31-(0)43-3882916  Medical Laboratory Dr. Stein & Collegae  Stadionplein 46  6225 XW Maastricht  The Netherlands  Tel: +31-(0)43-3622225 |
| **Pharmacy** | Not applicable |

**PROTOCOL SIGNATURE SHEET**

| **Name** | **Signature** | **Date** |
| --- | --- | --- |
| **Sponsor or legal representative:**  Not applicable  **For non-commercial research,**  **Head of Department:**  Prof. dr. Aalt Bast |  |  |
| **Coordinating Investigator/Project leader/Principal Investigator:**  Antje R. Weseler, PhD |  |  |

**TABLE OF CONTENTS**

1. INTRODUCTION AND RATIONALE [5](#__RefHeading___Toc302479310)

2. OBJECTIVES [8](#__RefHeading___Toc302479311)

3. STUDY DESIGN [9](#__RefHeading___Toc302479312)

4. STUDY POPULATION [11](#__RefHeading___Toc302479313)

4.1 Population (base) [11](#__RefHeading___Toc302479314)

4.2 Screening [11](#__RefHeading___Toc302479315)

4.3 Inclusion criteria [12](#__RefHeading___Toc302479316)

4.4 Exclusion criteria [12](#__RefHeading___Toc302479317)

4.5 Sample size calculation [13](#__RefHeading___Toc302479318)

5. TREATMENT OF SUBJECTS [14](#__RefHeading___Toc302479319)

5.1 Intervention [14](#__RefHeading___Toc302479320)

5.2 Use of co-intervention (if applicable) [14](#__RefHeading___Toc302479321)

5.3 Escape medication (if applicable) [14](#__RefHeading___Toc302479322)

6. INVESTIGATIONAL PRODUCT [15](#__RefHeading___Toc302479323)

6.1 Name and description of Investigational Substance [15](#__RefHeading___Toc302479324)

6.2 Description and justification of route of administration and dosage [15](#__RefHeading___Toc302479325)

7. METHODS [17](#__RefHeading___Toc302479326)

7.1 Study parameters/endpoints [17](#__RefHeading___Toc302479327)

7.1.1 Main study parameters/endpoints [17](#__RefHeading___Toc302479328)

7.1.2 Secondary study parameters/endpoints [17](#__RefHeading___Toc302479329)

7.1.3 Other study parameters [17](#__RefHeading___Toc302479330)

7.2 Randomisation, blinding and treatment allocation [18](#__RefHeading___Toc302479331)

7.3 Study procedures [18](#__RefHeading___Toc302479332)

7.3.1 Visits 2, 4 and 6 [19](#__RefHeading___Toc302479333)

7.3.2 Visits 3 and 5 [20](#__RefHeading___Toc302479334)

7.4 Withdrawal of individual subjects [20](#__RefHeading___Toc302479335)

7.4.1 Specific criteria for withdrawal [20](#__RefHeading___Toc302479336)

7.5 Replacement of individual subjects after withdrawal [20](#__RefHeading___Toc302479337)

7.6 Follow-up of subjects withdrawn from treatment [20](#__RefHeading___Toc302479338)

7.7 Premature termination of the study [21](#__RefHeading___Toc302479339)

8. SAFETY REPORTING [22](#__RefHeading___Toc302479340)

8.1 Section 10 WMO event [22](#__RefHeading___Toc302479341)

8.2 Adverse and serious adverse events [22](#__RefHeading___Toc302479342)

8.3 Follow-up of adverse events [22](#__RefHeading___Toc302479343)

9. STATISTICAL ANALYSIS [23](#__RefHeading___Toc302479344)

9.1 Descriptive statistics [23](#__RefHeading___Toc302479345)

9.2 Univariate analysis [23](#__RefHeading___Toc302479346)

9.3 Multivariate analysis [23](#__RefHeading___Toc302479347)

9.4 Interim analysis [23](#__RefHeading___Toc302479348)

10. ETHICAL CONSIDERATIONS [24](#__RefHeading___Toc302479349)

10.1 Regulation statement [24](#__RefHeading___Toc302479350)

10.2 Recruitment and consent [24](#__RefHeading___Toc302479351)

10.3 Objection by minors or incapacitated subjects (if applicable) [24](#__RefHeading___Toc302479352)

10.4 Benefits and risks assessment, group relatedness [24](#__RefHeading___Toc302479353)

10.5 Compensation for injury [26](#__RefHeading___Toc302479354)

10.6 Incentives (if applicable) [26](#__RefHeading___Toc302479355)

11. ADMINISTRATIVE ASPECTS AND PUBLICATION [27](#__RefHeading___Toc302479356)

11.1 Handling and storage of data and documents [27](#__RefHeading___Toc302479357)

11.2 Amendments [27](#__RefHeading___Toc302479358)

11.3 Annual progress report [27](#__RefHeading___Toc302479359)

11.4 End of study report [27](#__RefHeading___Toc302479360)

11.5 Public disclosure and publication policy [27](#__RefHeading___Toc302479361)

12. REFERENCES [28](#__RefHeading___Toc302479362)

**LIST OF ABBREVIATIONS AND RELEVANT DEFINITIONS**

| **Acaa** | Acetyl-CoA acyltransferase |
| --- | --- |
| **ACh** | Acetylcholine |
| **Acox** | Acetyl-CoA oxidase |
| **AE** | Adverse Event |
| **azM** | University Hospital Maastricht (in Dutch: akademisch ziekenhuis Maastricht) |
| **BMI** | Body Mass Index |
| **CA** | Competent Authority |
| **CCMO** | Central Committee on Research Involving Human Subjects (in Dutch: Centrale commissie mensgebonden onderzoek) |
| **CI** | Confidence interval |
| **CoA** | Certificate of Analysis |
| **COX** | Cyclo-oxygenase |
| **CRF** | Case report form |
| **CV** | Curriculum Vitae |
| **CVD** | Cardiovascular diseases |
| **DBP** | Diastolic blood pressure |
| **DNA** | Deoxyribonucleic acid |
| **Ech** | Enoyl-CoA hydratase |
| **EDTA** | Ethylendiaminetetraacetic acid |
| **FMD** | Flow mediated dilation |
| **GC-TOF-MS** | Gas chromatography time-of flight mass spectrometry |
| **HPLC** | High pressure liquid chromatography |
| **Hmgcs** | 3-hydroxy-3-methylglutaryl-CoA-synthase |
| **hsCRP** | High sensitivity C-reactive protein |
| **IB** | Investigator’s Brochure |
| **IC** | Informed Consent |
| **ICAM-1** | Intracellular adhesion molecule-1 |
| **IL** | Interleukin |
| **LDF** | Laser Doppler flowmetry |
| **L-NMMA** | N^G^-monomethyl-L-arginine |
| **Lpl** | Lipoproteinlipase |
| **MAP** | Mean arterial pressure |
| **METC** | Medical research ethics committee (MREC); in Dutch: medisch ethische toetsing commissie (METC) |
| **MI** | Myocardial infarction |
| **NO** | Nitric oxide |
| **NSAID** | Non-steroidal anti-inflammatory drug |
| **OPCs** | Oilgomeric proanthocyanidins |
| **Pcca** | Propionyl-CoA carboxylase |
| **Ph.Eur.** | European Pharmacopoeia |
| **rm ANOVA** | One-way repeated measures analysis of variance |
| **ROS** | Reactive oxygen species |
| **rpm** | rounds per minute |
| **s** | second |
| **(S)AE** | Serious Adverse Event |
| **SBP** | Systolic blood pressure |
| **SD** | Standard deviation |
| **SNP** | Sodium nitroprusside |
| **TEAC** | Trolox Equivalent Antioxidant Capacity |
| **TNF-α** | Tumour necrosis factor alpha |
| **VCAM-1** | Vascular cell adhesion molecule-1 |
| **Wbp** | Personal Data Protection Act (in Dutch: Wet Bescherming Persoonsgevens) |
| **WMO** | Medical Research Involving Human Subjects Act (Wet Medisch-wetenschappelijk Onderzoek met Mensen) |

**SUMMARY**

**Rationale:** Atherosclerosis and the associated complications of coronary artery disease and myocardial infarction (MI) account for the majority of the morbidity and mortality associated with cardiovascular disease (CVD). Smoking has been identified as a key risk factor for the development of CVD. It was found that a persistent increase in levels of oxidative stress and a prolonged inflammatory state play a pivotal role in the pathogenesis of smoking associated CVD. Oligomeric proanthocyanidins (OPC) are widely known for their anti-oxidant and anti-inflammatory effects, in vitro and in vivo. However, there are hardly any studies available that systematically investigated their acute and long-term effects on macro- and microvascular function as well as on established biomarkers of oxidative stress and inflammation in an “at risk” population such as smokers.

**Objective**: To evaluate the potential attenuating effects of an eight-week supplementation with 200 mg OPCs per day on macro- and microvascular function as well as on systemic biomarkers of inflammation and oxidative stress in healthy smoking subjects.

**Study design:** Double blind, randomised, placebo-controlled study with two parallel test groups.

**Study population:** 34 male smoking subjects aged between 30 - 60 years

**Intervention (if applicable)**: Subjects will be allocated randomly to one of the 2 test groups under accounting for their smoking habits. Subjects are asked to take either 2 capsules of the verum each containing 100 mg of the standardized OPC extract (MASQUELLIER’S® Original OPCs) or 2 capsules of the appropriate placebo product every morning over the 8 weeks intervention period.

**Main study parameters/endpoints:**

Main study parameter will be the improvement of the vasoreactivity of conduit and capillary arteries in smokers upon the intervention assessed by means of flow mediated dilation (FMD) (conduit arteries) and Laser Doppler flowmetry (LDF) after local administration of acetylcholine (ACh), coadministration of ACh and N^G^-monomethyl-L-arginine (L-NMMA) administration of sodium nitroprusside (SNP) by iontophoresis (microvasculature).

In addition, the following secondary study parameters will be assessed:

1. Plasma nitrite and nitrate levels
2. Systemic oxidative stress markers:
   1. Plasma levels of 8-iso-prostaglandin F2 (PGF2)α
   2. TEAC plasma levels
   3. GSH levels in erythrocytes
   4. Oxidative DNA damage in peripheral lymphocytes
   5. Gene expression levels of the redox enzymes superoxide dismutase, glutathione peroxidase, catalase, hemeoxygenase-1 in whole blood.
3. Systemic inflammation markers:
   1. Plasma levels of hsCRP
   2. Plasma levels of fibrinogen
   3. Plasma levels of inflammatory cytokines TNF-α, IL-1, IL-6, IL-8 and IL-10.
   4. Concentrations of the cytokines TNF-α, IL-1, IL-6, IL-8 and IL-10 after ex-vivo stimulation with lipopolysaccharide (LPS) of whole blood.
   5. Gene expression levels of cytokines (e.g. TNF-α, IL-1, IL-6, IL-8, IL-10), adhesion proteins (ICAM‑1), and I-kB-α in whole blood.

**Nature and extent of the burden and risks associated with participation, benefit and group relatedness:**

Subjects have to take 2 capsules of either the verum or the placebo every morning during an 8 week period. They will participate in 6 visits (including the screening visit) at the investigational site. Two visits will take approximately 30 min, three visits will last ca. 2.5 h, and the screening visit will take approximately 45 min.

Since individual acute benefits of the verum supplementation can hardly be predicted, and all subjects will undergo the same procedures, there is no real advantage or disadvantage of the allocation to one or the other test group.

In general, the risks associated with the participation are addressed and judged as follows:

1. Intake of investigational products

The intake of the OPCs is generally well-tolerated and not associated with any side effects. Moreover, both the placebo and the verum product will be delivered in pharmaceutical quality.

1. Blood collection

The risks being associated with the collected amount of 132 mL venous blood (screening visit ca. 12 mL and 3 study visits in 4 week intervals samplings of 40 mL per visit) are negligible.

1. LDF with iontophoresis of ACh, ACh and L-NMMA, and SNP

This is a slightly invasive, pain- and harmless technique to assess capillary blood flow. The vasoreactive substances ACh, L-NMMA and SNP will be delivered locally in quite low doses by means of a very low current. The compounds remain in the skin and do not become systemically available. Side effects are very seldom and can include allergic reactions. The laser used on the skin for the blood flow assessments does not elicit any side effects.

1. FMD measurements

Brachial artery flow-mediated dilation (FMD) is a non-invasive, pain- and harmless method to assess endothelium function of conduit arteries. Inflation of the forearm cuff may possibly cause transient ischemia of the subject’s arm which is generally not associated with any symptoms except of a potential slightly tingly sensation.

# INTRODUCTION AND RATIONALE

Cardiovascular disease (CVD) is the leading cause of death in the Western world. Atherosclerosis and the associated complications of coronary artery disease and myocardial infarction (MI) account for the majority of the morbidity and mortality associated with CVD. There is extensive experimental and clinical evidence for the pivotal role of persistently increased levels of oxidative stress and systemic inflammation within the manifestation and progression of atherosclerosis. Moreover, various epidemiological studies revealed strong associations between systemic biomarkers of oxidative stress and inflammation and an increased risk of CVD (1-3).

Chronic cigarette smoking is a major risk factor for CVD and is associated with endothelial dysfunction in coronary and peripheral conductance as well as resistance vessels (4-6). These changes in endothelial function are a crucial early step in the development of vascular diseases. However, the exact pathophysiological mechanisms linking smoking and endothelial dysfunction are complex and not fully elucidated, yet.

It is well established that cigarette smoke contains large quantities of free radical and pro-oxidant compounds (7). Moreover, it may indirectly increase free radical production by activation of macrophages and neutrophils, uncoupled endothelial nitric oxide synthase (eNOS), xanthine oxidase and mitochondrial electron transport chain (8-14). Interestingly, numerous studies found significantly elevated markers of systemic oxidative stress and inflammation in smokers (15-19) and at the same time depleted endogenous levels of antioxidants (7, 20). The obvious disturbance in the physiological redox state resulting in increased free radical-mediated oxidative stress can, therefore, largely explain the abnormalities in smokers occurring extrapulmonary in the vascular environment. Three integral components for the initiation and progression of vascular damage in smokers have been identified: (i) impairment in vasodilatory function, (ii) inflammation and (iii) modification of lipids.

The endothelium derived free radical nitric oxide (NO) is primarily responsible for the vasodilatory function of the endothelium. In vitro studies have shown that serum from smokers decreases the availability of NO by altering the expression and activity of eNOS (12, 21). In addition, NO may react with other radicals such as superoxide generating peroxynitrite, which further enhances the cellular oxidative stress (22). Since NO is not only a vasoregulatory molecule, but also helps to regulate inflammation, leukocyte adhesion, platelet activation and thrombosis, alterations in NO biosynthesis could have further consequences for processes involved in atherosclerosis and thrombosis formation and progression.

Cigarette smoking has been associated with increased levels of multiple inflammatory markers including CRP, interleukin (IL)-6, and tumour necrosis factor (TNF)-α (23-25). Moreover, elevated levels of various proinflammatory cytokines and adhesion molecules (such as soluble vascular cell adhesion molecule (VCAM)-1, intracellular adhesion molecule (ICAM)-1, P- and E-selectin) have been found in smokers implicating increased leukocyte recruitment and leukocyte-endothelial cell interactions (24, 26). In principle, smoking fuels the fire of inflammation in the blood and at the vessel wall (27).

Compared to non-smokers, smokers have significantly higher serum cholesterol, triglyceride, and low-density lipoprotein (LDL) levels, and lower serum concentrations of high-density lipoprotein (HDL) (28). In particular LDL is prone to oxidation due to the high levels of ROS and reactive nitrogen species present in smokers. Oxidatively modified LDL is taken up by macrophages, promoting cholesterol ester accumulation and foam cell formation, both being integral parts of atherosclerotic plaques. Basically, oxidised LDL as well as lipid peroxidation products are pro-inflammatory stimuli which further contribute to the manifestation of a low-grade systemic inflammation.

The central role of oxidative stress and inflammation in the pathogenesis of smoking associated cardiovascular diseases is additionally supported by studies which have shown that proatherogenic, proinflammatory and prothrombotic conditions of smokers can be improved or even reversed by agents that reduce oxidative stress or increase NO availability (12, 29-31). In search of such effective agents oligomeric proanthocyanidins (referred to as OPCs) display a promising class of compounds. Over the past years considerable evidence from epidemiological surveys could be gained for their ability to reduce the risk of cardiovascular diseases in different populations (32-35). OPCs are an integral part of the human diet and found in several commonly consumed foods. Especially apples, grapes (seeds and skin), chocolate and red wine are very rich sources of OPCs. These polyphenolic molecules were first identified and studied in depth by Dr. Jack Masquelier of the University of Bordeaux, France, in the late forties of the last century.

OPCs are composed of oligomeric forms of 2 to 5 single flavan-3-ol (catechin) units. They are potent antioxidants and exert protective activity on vascular tissue, making them of great interest in nutrition and human health. In fact, the beneficial effect of red wine in what has been long termed as the ‘French paradox’, i.e. a relatively low incidence of cardiovascular diseases despite having a diet relatively rich in saturated fat, is attributed to its content of (epi)catechins and oligomeric proanthocyanidins (36, 37). Other studies have suggested a link between intake of OPCs and reduction in one or more markers of cardiovascular disease risk such as endothelial dysfunction (38), lipid levels (39) or blood pressure (40). However, using a single marker could result in under-estimation of the risk of CVD as with the case of using cholesterol screening that fails to detect 50% of subjects that later develop MI (2, 41). Additionally, short-term intervention studies that assess markers following intake of the test product/dietary agent do not address the potential long-term benefits of this agent in reducing risk of disease. Analyzing changes in the expression of genes that are involved in regulating levels of these markers could be one approach for testing long-term effects on disease risk reduction (42). To our knowledge, a well-designed, comprehensive, study is lacking, in which the effects of a polyphenolic intervention are simultaneously assessed on physiological function parameters and established cardiovascular health biomarkers (representing short term risk improvement) and gene expression profiles in relevant tissues (representing long term risk improvement).

Therefore, in the present study we aim to address both approaches: (i) the assessment of direct effects of an 8-week intervention with the standardized OPC extract MASQUELIER'S® Original OPCs on macro- and microvascular function a well as on established and validated biomarkers of oxidative stress and inflammation and (ii) the assessment of the OPCs intervention’s effects on long-term disease risk by analyzing gene-expression profiles in the systemic body compartment whole blood. Based on the characteristics given above smokers represent an ideal cardiovascular “at risk” population for the investigation of our projected study objectives. Finally, the study is also intended to serve as a potential model for designing future trials that will allow reliable conclusions on the effects of foods/food components in reducing long-term disease risks and thereby permit scientifically-substantiated “disease risk reduction claims” that meet the criteria of European Regulation 1924/2006/EC on nutrition and health claims made on foods.

# OBJECTIVES

**Primary Objective**:

1. The primary objective of the present study is to show that an 8-weeks daily intake of 200 mg oligomeric proanthocyanidins (MASQUELLIER’S® Original OPCs) is able to improve arterial and/or microvascular function. Arterial function will be assessed by means of flow mediated dilation (FMD) of the brachial artery. Endothelium-dependent and independent microvascular function will be measured by means of Laser Doppler flowmetry (LDF) combined with iontophoresis of acetylcholine ACh, a combination of ACh + N^G^-monomethyl-L-arginine (L-NMMA), and sodium nitroprusside (SNP).

**Secondary Objectives**:

The secondary objectives of the study are to assess the effects of the 8-week intake of 200 mg OPCs on

1. plasma nitrite and nitrate levels.
2. systemic oxidative stress markers such as 8-iso-prostaglandin F2 (PGF2)α and TEAC plasma levels, GSH levels in erythrocytes, oxidative DNA damage in lymphocytes and gene expression levels of redox enzymes.
3. systemic inflammation parameters such as plasma levels of hsCRP, fibrinogen, the inflammatory cytokines TNF-α, IL-1, IL-6 and IL-8 and their gene expression levels.
4. cytokine levels (TNF‑α, IL-6, IL-8 and IL-10) in whole blood stimulated with LPS ex vivo.

**Other Objectives**:

Other objectives of the study are to assess the effects of the 8-week intake of 200 mg OPCs on

1. markers of plasma lipid levels such as HDL, LDL, VLDL, total cholesterol and triglycerides
2. markers of fatty acid metabolism by means of gene expression of enzymes involved in fatty acid catabolism such Lpl, Hmgcs2, Ech1, Acaa2, Acox1, Pcca.
3. platelet reactivity
4. arginase activity in erythrocytes
5. plasma levels of OPCs and their metabolites
6. blood pressure

# STUDY DESIGN

The study is designed as a double-blind, randomized, placebo-controlled trial with two parallel test groups.

Prior to the start of the intervention 34 eligible smokers will be randomized to one of the two test groups under taking into account the number of cigarettes smoked per day.

After baseline measurements during the first study visit, subjects in both groups will be asked to take 2 capsules of either the verum or the placebo product every morning for a period of 8 weeks.

After 4 and 8 weeks of test product intake, the study parameters will be measured again. Every two weeks the subjects will be invited to the investigational site in order to check their health and wellbeing and to provide them with new investigational products.

For every subject the study will last in total 8 weeks and requires including the screening visit 6 visits at the investigational site (see Fig. 1).


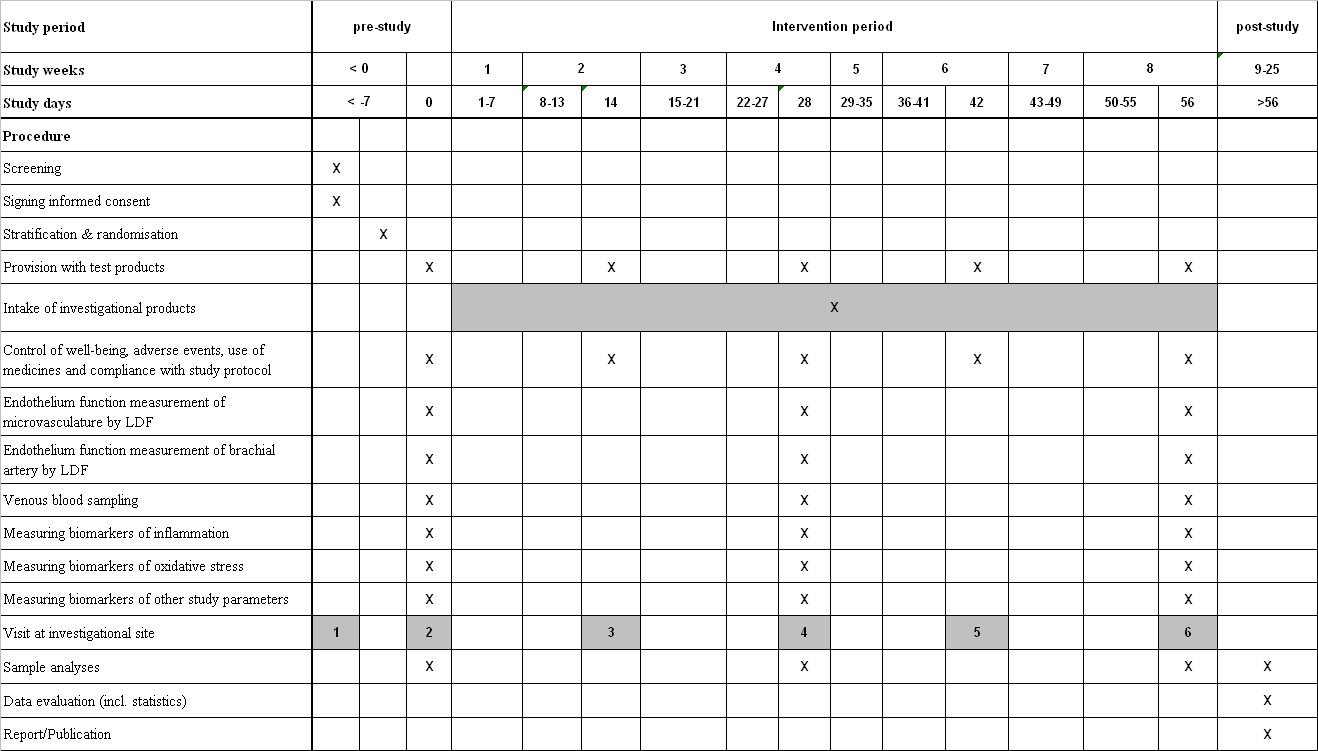
*Fig. 1: Flow -chart: Procedures and timelines*

# STUDY POPULATION

## Population (base)

The study population will comprise 34, 30-60 years old male smokers who are apparently healthy as assessed by the screening described in section 4.2.

The age range was set on base of the following considerations:

Men above 60 years with the projected smoking habits and history are expected to reveal increasing arterial stiffness and thus impaired vascular function (43, 44). This would considerably interfere with the detection of potential effects of the intervention.

On the other hand we consider the chance to recruit men below 30 years with the projected smoking habits and history as relatively small.

Women will not be eligible at all due to effects of female sex hormones on vasoreactivity and inflammatory processes (45-47).

## Screening

To verify whether potential subjects can be included in the study, a screening will be performed before the beginning of the trial. The screening involves the following measurements:

- Blood collection (ca. 12 mL in total) for
  - determination of a hemogram including white blood cells (WBC), red blood cells (RBC), platelets, haemoglobin, hematocrit
  - determination of blood glucose concentrations
  - control of standard clinical biomarkers for kidney (creatinine, Na^+^ and K^+^ concentrations) and liver function (alanine aminotransferase (ALAT), γ-glutamyl transferase (γ-GT) in plasma
  - control of acute inflammatory processes by measurement of plasma CRP concentrations
  - determination of total cholesterol, triglyceride, high density lipoprotein (HDL), low density lipoprotein (LDL) and very low density lipoprotein (VLDL) levels in serum
- Determination of weight and height, calculation of the BMI
- Measurement of blood pressure
- Assessment of the subject’s medical situation in present and past by a medical questionnaire including questions about lifestyle

The results of the measurements of the physical parameters as well as the results of the interview (documented in a standardized form) will be checked on the inclusion and exclusion criteria (see paragraph 4.3 and 4.4).

Finally, the responsible medical doctor will control every complete subject’s file on eligibility. When the subject is eligible, the responsible medical doctor will sign the eligibility form before the start of the study.

## Inclusion criteria

To become included to the trial the subjects have to comply with the following criteria:

- Male subjects in an age of 30-60 years
- smoking of 10 and more cigarettes/d and a regular smoking history of ≥ 5 years
- BMI ≥ 20 and ≤ 27 kg/m^2^
- Normal hemogram and normal blood levels of glucose, creatinine, albumin, ALAT, gamma-GT, AP, bilirubin (total)
- No reported acute and/or chronic inflammatory condition such as arthritis, arthrosis, chronic colitis etc. and/or indicated by a CRP > 3.0 mg/dl
- Serum cholesterol levels ≤ 7 mmol/L and triglycerides ≤ 2.5 mmol/L
- No reported physical and/or mental disease(s) or major surgery that might limit participation in or completion of the study
- No reported malignancy
- No reported current or previous metabolic (e.g. diabetes type I/II), cardiovascular and/or renal diseases
- No use of any medication that is known to influence lipid and glucose metabolism as well as blood pressure from at least one month before beginning of the study
- During the month prior to the start of the study no use of the following medications
  - antibiotics
  - local and systemic steroidal (glucocorticoids) and non-steroidal anti-inflammatory drugs (NSAID)
  - statines
- No excessive alcohol consumption (< 28 consumptions (approx. 250 g alcohol) per week)
- Normal constant eating habits during at least the last 3 months:
- No use of supplements, functional foods and/or other products containing vitamins, antioxidants and polyphenolic compounds or other ingredients with potential influence on vessel function for at least one month before the beginning of the study and during the entire study
- No use of a medically prescribed diet or slimming diet
- No vegetarian or vegan lifestyle
- No participation in a clinical trial within 4 weeks before the study

## Exclusion criteria

Subjects may be excluded from the study if they do not meet the inclusion criteria aforementioned.

In addition, during the execution of the study the following events and items can result in an exclusion:

- Intolerance of investigational products
- Occurrence of a (serious) adverse event (see paragraphs 7.2 and 7.3), in particular those which require the use of
  - Laxatives, anti-diarrhoeal drugs and any other medication that might influence the uptake of the investigational product
  - Antibiotics
  - Local and systemic steroidal (glucocorticoids) and non-steroidal anti-inflammatory drugs (NSAID)
  - Statines
  - Blood pressure lowering/elevating drugs
  - Drugs with influence on lipid and glucose metabolism
- Excessive alcohol consumption (> 28 consumptions (approx. 250 g alcohol) per week)
- Deviating from their usual lifestyle and physical activity habits

## Sample size calculation

There are currently no suitable data available that allow a persuasive sample size calculation for the effects of the 8 weeks consumption of the OPC extract assessed by either FMD or LDF. Due to this limitation the present study is considered as a pilot study to proof the concept and provide - among others - data for a power calculation required for a larger intervention study.

However, by recruiting the initially projected 34 subjects (17 subjects per test group under taking into account a drop-out rate of 10 %) and assuming a variance (σ) of 1.8 % for the change in brachial FMD (calculated as the change from baseline in diastolic diameter divided by the change from baseline in mean blood flow velocity), we are able to detect a change of 1.4 % in brachial FMD in a group of n = [2 σ (z_α_ + z_β_)^2^]/(µ_x_ - µ_y_)^2^ = 15 subjects with a power of 80% (β = 0.20; z_β_ = 0.84) and an α-value of 0.05 (z_α_ = 1.96) upon the OPC treatment.

# TREATMENT OF SUBJECTS

## Intervention

Subjects will be asked to take 2 capsules/d containing either 100 mg MASQUELIER’s Original OPCs or the equivalent amount of mircocristalline cellulose (placebo) over the 8 weeks intervention period every morning before their breakfast with a glass of water.

## Use of co-intervention (if applicable)

During the intervention subjects are requested not to change their usual dietary habits. The use of any functional foods/ food supplements or other products containing vitamins, antioxidants, polyphenols is not allowed during the trial (see also in- and exclusion criteria (chapter 4.3 and 4.4).

## Escape medication (if applicable)

Not applicable.

# INVESTIGATIONAL PRODUCT

## Name and description of Investigational Substance

The Investigational Substance to be used in the current study is MASQUELIER’s® Original OPCs. MASQUELIER’s® Original OPCs is extracted from the seeds of *Vitis vinifera* grapes, and contains catechins and epi-catechins (single flavan-3-ols) and OPCs (oligomeric flavan-3-ol units; dimers to pentamers). Detailed analyses have established that the product is standardized to contain about 85% (w/w) flavan-3-ols (as established with the Vanillin-H_2_SO_4_ assay (48)) of which 50-60% (w/w) are single and dimeric flavan-3-ols (determined by HPLC) and void of polymeric proanthocyanidins. The Investigational Substance will be applied in capsules each containing 100 mg of MASQUELIER'S® Original OPCs.

Moreover, appropriate placebo capsules will be manufactured that only contains the bulk ingredient microcrystalline cellulose.

## Description and justification of route of administration and dosage

**Route of administration of Investigational Product:**

The Investigational Substance MASQUELIER’s® Original OPCs has been widely available as an oral dietary supplement in several markets around the world since the late 1980s. The same active ingredient has been officially registered and sold as an oral herbal medicine in France under the trademark name Endotelon® since 1978. In all the human intervention studies described above, the subjects were administered MASQUELIER’s® Original OPCs orally, with significant biological efficacy and very minimal adverse effects (no serious adverse events and few minor events such as gastric discomfort and one case of vertigo). Based on this, it proposed that the subjects will be administered MASQUELIER’s® Original OPCs dietary supplement via the oral route in the current study.

**Dosage of Investigational Product:**

The dosage of the Investigational Product proposed to be used in the current study is 200mg/day. As various human intervention studies conducted with MASQUELIER’s® Original OPCs used doses ranging from 100mg/day to 450mg/day for biological effects in reducing veno-lymphatic insufficiency, vascular fragility, microcirculatory abnormalities in the eye, UV-induced inflammation and for improving visual adaptation to glare. These doses were found to be highly efficacious in the studies, and no major adverse events and very few minor events were reported in a total of 5536 subjects involved in the studies. Of the 5536 volunteers, only 1 subject dropped out of the studies due to adverse effects attributed to intake of Investigational Substance MASQUELIER’s® Original OPCs. This subject complained of vertigo, which resolved after discontinuing Investigational Substance intake (74). In animal studies, the Investigational Substance MASQUELIER’s® Original OPCs, at doses of 50 mg/kg given orally for 21 days, was found to effectively reduce vascular permeability in rats (51). No apparent toxicity was observed at these effective doses in this study. Conversion of the animal doses to human equivalent doses (HED) based on body surface area gives doses of 480 mg/day (US FDA CBER Guidance Document, 2003).

Based on these observations, we decided to use a dose of 200mg/day of Investigation Substance MASQUELIER’s® Original OPCs in the current study.

# METHODS

## Study parameters/endpoints

### Main study parameters/endpoints

The main study parameters in the present trial will be the improvement of the flow mediated dilation (FMD) of the brachial artery as well as the endothelium-dependent and ‑independent reactivity of microvasculature in smokers during an 8-week consumption of daily 200 mg oligomeric proanthocyanidins (MASQUELIER’S® Original OPCs). Microvascular function will be assessed by means of Laser Doppler flowmetry (LDF) after local application of ACh, a combination of ACh and L-NMMA and SNP by iontophoresis

### Secondary study parameters/endpoints

Moreover, the effects of the 8-week supplementation period with daily 200 mg OPCs or placebo will be investigated on the following secondary study parameters:

1. Plasma nitrite and nitrate levels
2. Systemic oxidative stress markers:
   1. Plasma levels of 8-iso-prostaglandin F2 (PGF2)α
   2. TEAC plasma levels
   3. GSH levels in erythrocytes
   4. Oxidative DNA damage in peripheral lymphocytes
   5. Gene expression levels of the redox enzymes superoxide dismutase, glutathione peroxidase, catalase, hemeoxygenase-1 in whole blood.
3. Systemic inflammation markers:
   1. Plasma levels of hsCRP
   2. Plasma levels of fibrinogen
   3. Plasma levels of inflammatory cytokines TNF-α, IL-1, IL-6, IL-8 and IL-10.
   4. Concentrations of the cytokines TNF-α, IL-1, IL-6, IL-8 and IL-10 after ex-vivo stimulation with lipopolysaccharide (LPS) of whole blood.
   5. Gene expression levels of cytokines (e.g. TNF-α, IL-1, IL-6, IL-8, IL-10), adhesion proteins (ICAM‑1), and I-kB-α in whole blood.

### Other study parameters

1) Plasma lipid levels:

- 1. High density lipoprotein (HDL)
  2. Low density lipoprotein (LDL)
  3. Very low density lipoprotein (VLDL)
  4. total cholesterol
  5. triglycerides

1. Gene expression levels of enzymes involved in fatty acid catabolism:
   1. Lipoproteinlipase (Lpl)
   2. 3-hydroxy-3-methylglutaryl-CoA-synthase 2 (Hmgcs2)
   3. Enoyl-CoA-hydratase (Ech1)
   4. Acetyl-CoA acyltransferase 2 (Acaa2)
   5. Acetyl-CoA oxidase 1 (Acox1)
   6. Propionyl-CoA carboxylase (Pcca)
2. Platelet reactivity
3. Arginase activity in erythrocytes
4. Plasma concentrations of OPCs and their metabolites
5. Blood pressure

## Randomisation, blinding and treatment allocation

We will allocate the subjects to one of the two treatments in a random manner. Since the amount of cigarettes smoked per day directly reflects the subject’s level of oxidative stress (7), we will balance the groups with respect to the individual smoking habits. This will be achieved by classifying smoking habits in the following manner:

1. 10 – 20 cigarettes per day
2. > 20 cigarettes per day

The study will be carried out in a double-blind fashion, i.e. neither the subject nor the investigators will be aware of the treatment a subject receives.

The identity of the investigational products will be blinded by a numerical code.

The capsules will be packaged in suitable blisters containing the appropriate number of capsules for a 2-week intervention (i.e. 2 x 14 = 28 capsules) of an individual subject. The blisters will be packed in small boxes properly labelled with a unique numerical code.

In addition, three identical envelopes will be prepare each containing again two envelopes with the respective treatment codes.

One envelop will be stored at a secured place where the principal investigator will have direct access; another envelop will be stored at a secured place where the responsible medical doctor will have direct access, and the third envelop will be kept at a secured place at the head of the Toxicology Department’s site.

As soon as a serious adverse event (SEA) will occur during the study period (for definition see chapter 8.2), the responsible medical doctor and the principal investigator are ultimately responsible for the final decision on breaking the code. Only in case of medical urgency, the blinding code can be broken.

If it does not become necessary to break the blinding codes throughout the study, decoding will first take place after a blind review including preliminary statistical analyses of the data of the primary study parameter assessed by the investigators.

In all cases, unblinding will take place under survey of a witness.

## Study procedures

After signing of the informed consent form before the screening (visit 1), subjects will be invited for further five test sessions at the investigational site.

The test sessions will take place prior to the beginning of the intervention period (visit 2), and after two (visit 3), four (visit 4), six (visit 5) and eight (visit 6) weeks of intervention (see also fig 1).

### Visits 2, 4 and 6

These sessions will be carried out in the Circulation Lab of the Dept. of Internal Medicine, MUMC under supervision of experienced lab assistants and the principle investigator. The lab has gained extensive experience in the performance of forearm blood flow measurements over the past years (76-83).

Subjects will be asked to refrain from smoking and beverages containing caffeine or alcohol for at least 12 hours before the experiments, which will be scheduled at around 8 a.m. after an overnight fast (i.e. no consumption of food and drinks later than 22.00h the evening before the test day except of drinking water).

After arrival in the lab, subjects’ well-being will briefly be checked as well as the occurrence of any adverse event, the use of concomitant medication and compliance to the study protocol based on the notes in their study diary. Consequently, the subjects will receive the Investigational Products for the next 2 weeks (not applicable on visit 6) and the measurements will commence.

The measurements will be carried out in a quite room with a constant temperature of 23°C. The subjects will be studied in the supine position and remain in bed during the experiments. Prior to the vascular function measurements blood pressure will be measured by a suitable automatic device and according to the standard procedure of the Circulation Lab.

1. **Microvascular function measurement**

By means of LDF the superficial skin perfusion will be assessed in the finger before and after local application of ACh (1% Miochol, IOLAB, Bourneville Pharma, Netherlands), a mix of ACh (1%) and L-NMMA (1% Clinalfa, Bachem, Switzerland) and SNP (0.01 Nipride, Roche, Netherlands) via iontophoresis and in accordance with a standard protocol (84, 85).

By means of a low positive current (0.1 mA for 20 s) ACh will migrate across the skin. Seven increasing doses of ACh will be applied locally with an interval of 60 s between each dose.

After the last ACh measurement the LDF probe will relocated slightly on the skin to prevent, that potential rests of ACh may influence the measurements with SNP.

Coadministration of ACh and L-NMMA will be done in the same way as described for the ACh application.

SNP will locally delivered by means of a low negative current (0.2 mA for 20 s) in seven increasing doses with an interval of 90 s between each dose.

The ACh and the ACh + L-NMMA measurements will take approximately 10 min.; the SNP measurements will last approximately 15 min.

During all measurements skin temperature will continuously be monitored and regulated by the Laser Doppler probe. The skin temperature has to remain above 28°C.

1. **Conduit artery function measurement**

Endothelial function of conduit vessel will be measured in the bracherial artery of the non-dominant arm, just above the elbow (86, 87). By means of a vessel wall-movement detector system consisting of an ultrasound imager connected to a data acquisition and processing unit arterial diameter and peak flow velocity will be recorded for 3 min. at baseline.

Consequently, a pressure cuff placed around the forearm will be inflated to a supra-systolic pressure (systolic pressure + 40 mmHg) in order to induce forearm ischemia. After 5 min. of sustaining this pressure the cuff will be released. As a consequence blood flows with an increased velocity in the vessel causing an increased shear stress on the endothelium which serves as the stimulus for FMD. The arterial diameter and peak flow velocity will be measured continuously after cuff release for further 240 s. The relative increase in diameter related to the evoked relative increase in blood flow will be used in the statistical analysis (88).

The FMD measurement will take approximately 15 min.

The entire test session will last approximately 2.5 hours in total.

### Visits 3 and 5

For these visits it is not required that the subjects are in a fasted state, because it is not intended to assess any of the study parameters on these occasions. The visits are rather aimed to control the subject’s well-being, the experience of any adverse events, the use of concomitant medication, the compliance with the intake of the investigational product and other relevant study procedures. Also notes in the study diary (see annex 1) will be considered in the discussion. At the end the subjects will receive the Investigational Product for the following 2 weeks.

These sessions will not exceed 30 min.

## Withdrawal of individual subjects

Subjects can leave the study at any time for any reason if they wish to do so without any consequences. The investigator can decide to withdraw a subject from the study for urgent medical reasons (see chapter 4.4).

### Specific criteria for withdrawal

See chapter 4.4

## Replacement of individual subjects after withdrawal

Subjects who withdraw during the first two weeks will try to be replaced. Subjects who will drop-out during a later time in the trial will not be replaced.

## Follow-up of subjects withdrawn from treatment

When a subject is withdrawn prematurely from the study, proper follow up will be given if necessary.

## Premature termination of the study

In case of premature termination/suspension of the trial for any reason, the investigator will inform:

- the regulatory authorities (if applicable)
- the trial subject, assuring him/her appropriate treatment and follow up

If the investigator terminates or suspends the trial without prior agreement of the sponsor, then the institution should:

- promptly inform and provide the sponsor and the METC with a detailed written explanation of the termination or suspension.

If the sponsor terminated/suspends the trial, then the institution will:

- promptly inform and provide the METC with detailed written explanation of the termination or suspension.

If the METC terminates or suspends its approval of the trial, then the institution will:

- promptly notify and provide the sponsor with a detailed written explanation of the termination or suspension.

# SAFETY REPORTING

## Section 10 WMO event

In accordance to section 10, subsection 1, of the WMO, the investigator will inform the subjects and the reviewing accredited METC if anything occurs, on the basis of which it appears that the disadvantages of participation may be significantly greater than was foreseen in the research proposal. The study will be suspended pending further review by the accredited METC, except insofar as suspension would jeopardise the subjects’ health. The investigator will take care that all subjects are kept informed.

## Adverse and serious adverse events

An adverse event is defined as any undesirable experience occurring to a subject during a clinical trial, whether or not considered related to the clinical trial.

The medical occurrence is untoward in frequency and/or kind. An Adverse Event can be any unfavourable or unintended phenomenon (including abnormal laboratory values), every symptom that or every illness that is time-related to the use of an investigational product, either or not related to the intake of this investigational product.

At every study visit the subjects will be asked about their well-being and the experience of any adverse event.
Details of all adverse events reported spontaneously by subjects or observed by the investigator or medical staff will be recorded in appropriate adverse event report forms and reported to the principal investigator and the responsible medical doctor. All adverse events observed will be recorded separately.

A serious adverse event is any untoward medical occurrence or effect that at any dose results in death:

- is life threatening (at the time of the event);
- requires hospitalisation or prolongation of existing inpatients’ hospitalisation;
- results in persistent or significant disability or incapacity;
- is a new event of the trial likely to affect the safety of the subjects, such as an unexpected outcome of an adverse reaction, lack of efficacy of an IMP used for the treatment of a life threatening disease, major safety finding from a newly completed animal study, etc.

All SAEs will be reported to the METC of Maastricht University, which approved the protocol, according to the requirements of the METC.

## Follow-up of adverse events

All adverse events will be followed until they have abated, or until a stable situation has been reached. Depending on the event, follow up may require additional tests or medical procedures as indicated, and/or referral to the general physician or a medical specialist.

# STATISTICAL ANALYSIS

## Descriptive statistics

All normally distributed data will be presented as mean ± standard deviation (SD). If data are not normally distributed the median and 25^th^-75^th^ percentiles will be given.

This approach will be applied to the baseline characteristics of the study population as well as to the primary and secondary study parameters.

## Univariate analysis

Differences in baseline characteristics between the subjects will be assessed by a parametric test (independent-samples Student’s t-test), if data are normally distributed. In case of non-normality, data will be transformed to obtain a normal distribution. However, if this transformation will not be successful, differences will be tested by the nonparametric Mann-Whitney U-test.

**Evaluation of the vascular function parameters**

Differences in capillary blood flow (before and after delivery of ACh and SNP, respectively) and brachial artery FMD (calculated as the change from baseline in diastolic diameter divided by the change from baseline in mean blood flow velocity) between the two test groups will be tested at each time point by independent-samples Student’s t-test (if normally distributed) or Mann-Whitney-U-Test (in case of non-normal distribution and unsuccessful transformation).

**Other study parameters**

The individual changes from baseline in each of the other parameters (secondary study parameters # 4-6 and other study parameters # 1-4) assessed will be calculated separately for the 4-week intervention interval and the 8-week intervention interval. If data are normally distributed, significant different alterations between the verum and the placebo group will be appraised per study parameter by independent-samples Student’s t-test.

In case data will not be distributed normally, transformation will be performed to achieve normal distribution. If transformation remains unsuccessful, Mann-Whitney U-test will be used for the analyses.

All tests will be carried out two-sided. Statistical significance will be accepted at the 95% confidence level (p ≤ 0.05).

## Multivariate analysis

Multivariate analyses are not intended to be performed.

## Interim analysis

Interim analyses are not intended to be performed.

# ETHICAL CONSIDERATIONS

## Regulation statement

The study will be conducted according to the ethical principles of the Declaration of Helsinki, revision 2000. Before the study will start, the protocol and all relevant documents will be approved by the Medical Ethics Committee of the University Hospital Maastricht and Maastricht University.

## Recruitment and consent

Subjects will be recruited in Maastricht and its vicinity by advertisements in local weekly newspapers as well as by posters and flyers on bulletin boards within the Maastricht university building, the Maastricht academic hospital (azM) and other public places.

Interested subjects will receive oral (by telephone call) and written information (see enclosed Information Brochure and Informed Consent Form, in Dutch) about the study. They can read these documents quietly at home and can contact the investigators by phone and/or email at any time to receive further details about the study and to ask all their questions.

The subject will have one week to come to his decision on his participation. If he decides to participate he will be asked to sign the Informed Consent Form and to provide this form the investigators at the beginning of the screening visit (visit 1).

Subjects will voluntarily sign the Informed Consent Form in which they agree to participate in the study and in which they confirm that they have been properly informed about the study.

After signing the Informed Consent Form the subject is allowed to withdraw from the study at any stage without giving any reason.

Information and appointments before and during the study will be provided in written by means of the Information Brochure and a small study diary in which subjects can daily note any remarks with respect to the intake of the test products, their well-being, deviations from their usual use of medication and any adverse events.

Subjects who wish to receive more information about the trial can also contact the independent medical doctor Dr. Wierema. He is fully informed about the study, but he is not involved in the trial.

After participation, subjects may receive their personal results of the screening. If they wish, they may receive a summary of the general outcomes of the study after finalization of data analyses and evaluation.

## Objection by minors or incapacitated subjects (if applicable)

Not applicable.

## Benefits and risks assessment, group relatedness

Direct advantages of a subject’s participation in the study are hardly to predict. However, the findings of the study will considerably contribute to the elucidation of the anti-oxidant, anti-inflammatory and vascular properties of this well-characterised OPC extract in a smokers, a population with increased oxidative stress and risk of CVD. Moreover, by simultaneously analyzing gene-expression profiles in relevant tissues we seek to address the effects of the OPC intervention on a long-term risk of disease.

Participation in the trial might be associated with several risks being addressed and judged as follows:

1. Intake of investigational products

The intake of the OPCs is generally well-tolerated and not associated with any side effects (see also chapter 5.4). Moreover, both the placebo and the verum product will be delivered in pharmaceutical quality, i.e. identity, purity and quantity will be assured for both, the raw materials and the application form, i.e. the capsules prepared (see also appendices A1-A8).

1. Blood collection

As a consequence of the intra-venous punctures local haematomas can occur. However, most of the times this is a transient local reaction which usually does not require any medical care.

The risks being associated with the collected amount of 132 mL venous blood in total (screening visit ca. 12 mL and 3 study visits in 4 week intervals samplings of 40 mL per visit) are negligible.

1. LDF with iontophoresis of ACh and SNP

LDF with iontophoresis is a slightly invasive, pain- and harmless technique to assess capillary blood flow. The vasoreactive substances ACh and SNP (for detailed information see Appendix A9 & A10) will be delivered locally in quite low doses by means of a very low current. The compounds remain in the skin and do not become systemically available. Side effects are very seldom and can include allergic reactions.

The laser used on the skin for the blood flow assessments does not elicit any side effects.

1. FMD measurements

Brachial FMD measurement is a non-invasive, pain- and harmless method to assess endothelium function of conduit arteries (87). Inflation of the forearm cuff may possibly cause transient ischemia of the subject’s arm which is generally not associated with any symptoms except of a potential slightly tingly sensation.

## Compensation for injury

Maastricht University has insurance for all subjects as required. The insurance company is Marketform/Lloyd’s London. The insurance is an insurance for studies at standard risk (see enclosed insurance declaration, in Dutch).

This insurance provides cover for damage to research subjects through injury or death caused by the study.

1. € 450.000,-- (i.e. four hundred and fifty thousand Euro) for death or injury for each subject who participates in the Research;
2. € 3.500.000,-- (i.e. three million five hundred thousand Euro) for death or injury for all subjects who participate in the Research;
3. € 5.000.000,-- (i.e. five million Euro) for the total damage incurred by the organisation for all damage disclosed by scientific research for the Sponsor as ‘verrichter’ in the meaning of said Act in each year of insurance coverage.

The insurance applies to the damage that becomes apparent during the study or within 4 years after the end of the study.

Maastricht University has a liability insurance in place, as required.

## Incentives (if applicable)

All subjects will receive a financial compensation of € 100,- after completion of 4 weeks intervention and € 200,- after having finalized the entire study.

Travel costs will be reimbursed for all subjects on the basis of public transport 2^nd^ class.

If a subject withdraws from the study prematurely, the compensation will be paid out pro-rata.

# ADMINISTRATIVE ASPECTS AND PUBLICATION

## Handling and storage of data and documents

To guarantee their privacy, at the start of the study all subjects will receive a subject trial number consisting of a 3-digit code. Only the main investigator (Dr. A. Weseler), a supporting investigator (to be appointed, yet) and the responsible medical doctor (Dr. G. Koek) are authorized to have access to the personal subject information.
Basically, all data will be made anonymous before transferred to third parties or published.

Raw data will be collected on paper and will be made electronic available. The electronic entered data will be checked by a second person on correctness and completeness. The paper data sheets as well as the electronic data files will be archived at the investigational site according to the regulatory requirements until 2023.

## Amendments

Amendments are changes made to the research after a favourable opinion by the accredited METC has been given. All amendments will be notified to the METC that gave a favourable opinion.

## Annual progress report

The investigator (sponsor) will submit a summary of the progress of the trial to the accredited METC once a year. Information will be provided on the date of inclusion of the first subject, numbers of subjects included and numbers of subjects that have completed the trial, serious adverse events/ serious adverse reactions, other problems, and amendments.

## End of study report

The investigator (sponsor) will notify the accredited MEC on the end of the study within a period of 12 weeks (90 days). The end of the study is defined as the last visit of the last tested individual.

Information will be provided on the date of inclusion of the first subject, numbers of subjects included and numbers of subjects that have completed the trial, serious adverse events/ serious adverse reactions, other problems, and amendments

In case the study is ended prematurely, the investigator (sponsor) will notify the accredited MEC (and the competent authority) within 15 days, including the reasons for the premature termination.

## Public disclosure and publication policy

It is intended to publish the results immediately in an appropriate peer-reviewed scientific journal upon completion of the sample analyses and data evaluation.

# REFERENCES

1. Libby P, Ridker PM. Novel Inflammatory Markers of Coronary Risk : Theory Versus Practice. Circulation 1999;100:1148-1150.

2. Rifai N, Ridker P. High-sensitivity C-reactive protein: a novel and promising marker of coronary heart disease. Clin Chem 2001;47:403-411.

3. Ross R. Atherosclerosis - an inflammatory disease. N Engl J Med 1999;340:115-126.

4. Howard G, Wagenknecht L, Burke G, et al. Cigarette smoking and progression of atherosclerosis: the atherosclerosis risk in communities (ARIC) study. JAMA 1998;279:119-124.

5. Leone A. Relationship between cigarette smoking and other coronary risk factors in atherosclerosis: risk of cardiovascular disease and preventive measures. Curr Pharm Des 2003;9:2417-2423.

6. Teo K, Ounpuu S, Hawken S, et al. Tobacco use and risk of myocardial infarction in 52 countries in the INTERHEART study: a case-control study. Lancet 2006;368:647-658.

7. Yanbaeva DG, Dentener MA, Creutzberg EC, Wesseling G, Wouters EFM. Systemic Effects of Smoking. Chest 2007;131:1557-1566.

8. Pryor WA, Stone K. Oxidants in cigarette smoke. Radicals, hydrogen peroxide, peroxynitrate, and peroxynitrite. Ann N Y Acad Sci 1993;686:12-27; discussion 27-8.

9. Pryor WA, Stone K, Zang LY, Bermudez E. Fractionation of aqueous cigarette tar extracts: fractions that contain the tar radical cause DNA damage. Chem Res Toxicol 1998;11:441-8.

10. Powell JT. Vascular damage from smoking: disease mechanisms at the arterial wall. Vascular Medicine 1998;3:21-28.

11. Smith CJ, Fischer TH. Particulate and vapor phase constituents of cigarette mainstream smoke and risk of myocardial infarction. Atherosclerosis 2001;158:257-267.

12. Barua RS, Ambrose JA, Srivastava S, DeVoe MC, Eales-Reynolds L-J. Reactive Oxygen Species Are Involved in Smoking-Induced Dysfunction of Nitric Oxide Biosynthesis and Upregulation of Endothelial Nitric Oxide Synthase: An In Vitro Demonstration in Human Coronary Artery Endothelial Cells. Circulation 2003;107:2342-2347.

13. Guthikonda S, Sinkey C, Barenz T, Haynes WG. Xanthine Oxidase Inhibition Reverses Endothelial Dysfunction in Heavy Smokers. Circulation 2003;107:416-421.

14. Heitzer T, Brockhoff C, Mayer B, et al. Tetrahydrobiopterin Improves Endothelium-Dependent Vasodilation in Chronic Smokers : Evidence for a Dysfunctional Nitric Oxide Synthase. Circ Res 2000;86:e36-41.

15. Morrow JD, Frei B, Longmire AW, et al. Increase in circulating products of lipid peroxidation (F2-isoprostanes) in smokers. Smoking as a cause of oxidative damage. N Engl J Med 1995;332:1198-203.

16. Orhan H, Evelo CT, Sahin G. Erythrocyte antioxidant defense response against cigarette smoking in humans--the glutathione S-transferase vulnerability. J Biochem Mol Toxicol 2005;19:226-33.

17. Miller LG, Goldstein G, Murphy M, Ginns LC. Reversible alterations in immunoregulatory T cells in smoking. Analysis by monoclonal antibodies and flow cytometry. Chest 1982;82:526-529.

18. Hughes DA, Haslam PL, Townsend PJ, Turner-Warwick M. Numerical and functional alterations in circulatory lymphocytes in cigarette smokers. Clin Exp Immunol 1985;61:459-66.

19. Bazzano LA, He J, Muntner P, Vupputuri S, Whelton PK. Relationship between Cigarette Smoking and Novel Risk Factors for Cardiovascular Disease in the United States. Ann Intern Med 2003;138:891-897.

20. Rahman I, Swarska E, Henry M, Stolk J, MacNee W. Is there any relationship between plasma antioxidant capacity and lung function in smokers and in patients with chronic obstructive pulmonary disease? Thorax 2000;55:189-193.

21. Barua RS, Ambrose JA, Eales-Reynolds L-J, DeVoe MC, Zervas JG, Saha DC. Dysfunctional Endothelial Nitric Oxide Biosynthesis in Healthy Smokers With Impaired Endothelium-Dependent Vasodilatation. Circulation 2001;104:1905-1910.

22. Kojda G, Harrison D. Interactions between NO and reactive oxygen species: pathophysiological importance in atherosclerosis, hypertension, diabetes and heart failure. Cardiovasc Res 1999;43:562-71.

23. Mendall MA, Patel P, Asante M, et al. Relation of serum cytokine concentrations to cardiovascular risk factors and coronary heart disease. Heart 1997;78:273-277.

24. Bermudez EA, Rifai N, Buring JE, Manson JE, Ridker PM. Relation between markers of systemic vascular inflammation and smoking in women. The American Journal of Cardiology 2002;89:1117-1119.

25. Tracy RP, Psaty BM, Macy E, et al. Lifetime Smoking Exposure Affects the Association of C-Reactive Protein with Cardiovascular Disease Risk Factors and Subclinical Disease in Healthy Elderly Subjects. Arterioscler Thromb Vasc Biol 1997;17:2167-2176.

26. Mazzone A, Cusa C, Mazzucchelli I, et al. Cigarette smoking and hypertension influence nitric oxide release and plasma levels of adhesion molecules. Clin Chem Lab Med 2001;39:822-6.

27. Ambrose JA, Barua RS. The pathophysiology of cigarette smoking and cardiovascular disease: An update. J Am Coll Cardiol 2004;43:1731-1737.

28. Craig WY, Palomaki GE, Haddow JE. Cigarette smoking and serum lipid and lipoprotein concentrations: an analysis of published data. Bmj 1989;298:784-8.

29. Heitzer T, Just H, Munzel T. Antioxidant Vitamin C Improves Endothelial Dysfunction in Chronic Smokers. Circulation 1996;94:6-9.

30. Fennessy FM, Moneley DS, Wang JH, Kelly CJ, Bouchier-Hayes DJ. Taurine and Vitamin C Modify Monocyte and Endothelial Dysfunction in Young Smokers. Circulation 2003;107:410-415.

31. Takajo Y, Ikeda H, Haramaki N, Murohara T, Imaizumi T. Augmented oxidative stress of platelets in chronic smokers: Mechanisms of impaired platelet-derived nitric oxide bioactivity and augmented platelet aggregability. J Am Coll Cardiol 2001;38:1320-1327.

32. De Lorgeril M, Salen P, Paillard F, Laporte F, Boucher F, De Leiris J. Mediterranean diet and the French paradox: two distinct biogeographic concepts for one consolidated scientific theory on the role of nutrition in coronary heart disease. Cardiovasc Res 2002;54:503-515.

33. Meydani M. Nutrition inteventions in aging and age-associated disease. Ann NY Acad Sci 2001;928:226-235.

34. Panagiotakos D, Sitara M, Pitsavos C, Stefanadis C. Estimating the 10-year risk of cardiovascular disease and its economic consequences, by the level of adherence to the Mediterranean diet: the ATTICA study. J Med Food 2007;10:239-243.

35. Thomas G, Cheung B, Ho S, et al. Overview of dietary influences on atheorsclerotic vascular disease: epidemiology and prevention. Cardiovasc Hematol Disord Drug Targets 2007;7:87-97.

36. Corder R, Mullen W, Khan N, et al. Oenology: red wine procyanidins and vascular health. Nature 2006;444:566.

37. Teissedre P, Cabanis J, Moutonnet M. Wine and health: the current picture. Wine and health - Guide, 1996.

38. Heiss C, Kleinbongard P, Dejam A, et al. Acute Consumption of Flavanol-Rich Cocoa and the Reversal of Endothelial Dysfunction in Smokers. Journal of the American College of Cardiology 2005;46:1276-1283.

39. Fraga C, Actis-Goretta L, Ottaviani J, et al. Regular consumption of a flavanol-rich chocolate can improve oxidant stress in young soccer players. Clin Dev Immunol 2005;12:11-17.

40. Grassi D, Lippi C, Nedozione S, Desideri G, Ferri C. Short-term administration of dark chocolate is followed by a significant increase in insulin sensitivity and a decrease in blood pressure in healthy persons. Am J Clin Nutr 2005;81:611-614.

41. Paramo J, Rodriguez J, Orbe J. Vulnerable plaque versus vulnerable patient: emerging blood biomarkers for risk stratification. Endocrin Metab Immune Disord Drug Targets 2007;7:195-201.

42. Afman L, Muller M. Nutrigenomics: from molecular nutrition to prevention of disease. J Am Diet Assoc 2006;106:569-76.

43. Mitchell GF, Parise H, Benjamin EJ, et al. Changes in Arterial Stiffness and Wave Reflection With Advancing Age in Healthy Men and Women: The Framingham Heart Study. Hypertension 2004;43:1239-1245.

44. Joseph P Noon TCTSAGSGRLS. The effect of age and gender on arterial stiffness in healthy Caucasian Canadians. Journal of Clinical Nursing 2008;9999.

45. Williams MRI, Westerman RA, Kingwell BA, et al. Variations in Endothelial Function and Arterial Compliance during the Menstrual Cycle. J Clin Endocrinol Metab 2001;86:5389-5395.

46. Bartelink ML, Wollersheim H, Theeuwes A, van Duren D, Thien T. Changes in skin blood flow during the menstrual cycle: the influence of the menstrual cycle on the peripheral circulation in healthy female volunteers. Clin Sci (Lond) 1990;78:527-32.

47. Gerhardt U, Hillebrand U, Mehrens T, Hohage H. Impact of estradiol blood concentrations on skin capillary Laser Doppler flow in premenopausal women. International Journal of Cardiology 2000;75:59-64.

48. Sun B, Ricardo da Silva J, Spranger I. Critical factors of vanillin assay for catechins and proanthocyanidins. J Agric Food Chem 1998;46:4267-4274.

49. Oligomeric proanthocyanidins. Monograph. Alternative Medicine Review 2003;8:442-450.

50. LaParra J, Michaud J, Masquelier J. Action des oligomeres procyanidoliques sur le cobaye carence en vitamine C. Bull Soc Pharm Bordeaux 1979;118:7-13.

51. Gavignet-Jeannin C, Groult N, Godeau G, Robert A, Robert L. Mode d'action des oligomeres procyanidoliques sur la paroi vasculaire. Congres International d'Angioloogie, symposium satellite Endotelon et unite circulatoire. Toulouse, 1988.

52. Wegrowski J, Robert A, Moczar M. The effect of procyanidolic oligomers on the composition of normal and hypercholesterolemic rabbit aortas. Biochem Pharmacol 1984;33:3491-3497.

53. Blazso G, Gabor M. Oedema-inhibiting effect of procyanidin. Acta Physiol Acad Sci Hung 1980;56:235-240.

54. Pfister A, Simon M, Gazave J. Sites de fixation des oligomeres procyanidoliques dans la paroi des capillaires sanguins du poumon de cobaye. Acta Therapeutica 1982;8:223-237.

55. Tixier J, Godeau G, Robert A, Hornebeck W. Evidence by in vivo and in vitro studies that binding of pycnogenols to elastin affects its rate of degradation by elastases. Biochem Pharmacol 1984;33:3933-3939.

56. Masquelier J, Dumon M, Dumas J. Stabilisation du collagene par les oligomeres procyanidoliques. Acta Therapeutica 1981;7:101-105.

57. Masquelier J. Effets physiologiques du vin - Sa part dans l'alcoolisme. Bulletin de l'O.I.V. 1988;689-690:555-578.

58. De Haan B, Bapat S, Post J. Protection of vascular endothelial cells from oxidative damage by oligomeric proanthocyanidins. XVI Congress of the oxygen club of California on oxidants and antioxidants in biology. Santa Barbara, CA, USA, 2006.

59. Meunier M, Duroux E, Bastide P. Activite antiradicalaire d'oligomeres procyanidolique et d'anthocyanosides vis-a-vis de l'anion superoxyde et vis-a-vis de la lipoperoxydation. Plantes medicinales et Phytotherapie 1989;13:267-274.

60. Barbier A, Maffrand J, Savi P, Unkovic J, Villain P. International Congress on Angiology in Toulouse. International Congress on Angiology. Toulouse, France, 1988:31-40.

61. Thebaut J-F, Thebaut P, Vin F. Etude de l'endotelon dans les manifestations functionelles de l'insufficisance veineuse peripherique. Resultats d'une etude en double aveugle portant sur 92 patients. Gazette Medicale 1985;92:96-100.

62. Delacroix P. Etude en double aveugle de l'endotelon dans l'insuffisance veineuse chronique. La Revue de Medecine 1981;22:1793-1802.

63. Elbaz C, Reinharez D, Sapin G, et al. Etude multicentrique controlee de l'endotelon dans les manifestations fonctionelles de l'insufficance veineuse chronique des membres inferieurs. Le Praticien 1981;400:63-68.

64. Sarrat L. Abord therapeutique des troubles fonctionelles des membres inferieurs par un microangioprotecteur l'endotelon. Bordeaux Medicale 1981;11:685-688.

65. Henriet J. Insuffisance veino-lymphatique 4729 patientes sous therapeutique hormonale et oligomeres procyanidoliqques. Phelobologie 1993;46:313-326.

66. Dartenuc J, Marache P, Choussat H. Resistance capillaire en geriatrie: etude d'un microangioprotecteur endotelon. Bordeaux Med 1980;13:903-907.

67. Dubos G, Durst G, Hugonot R. Evolution de la resistance capillaire, spontanement ou artificiellement diminuee par l'action d'un substance capillaro-toxique chez des personnes agees. La Revue de Geriatrie 1980;5:302-305.

68. Beylot C, Bioulac P. Essai therapeutique d'un angioprotecteur peripherique, l'endotelon. Gazette Medicale de France 1980;87:2919-2922.

69. Pecking A, Picandet B, Hacene K, Lokiec F, Guerin P. Oligomeres procyanidoliques (Endotelon) et systeme lymphatique. Arteres et Veines 1987;6:512-513.

70. Baruch J. Effet de l'endotelon dans les oedemes post-chirurgicaux. Ann Chir Plast Esthet 1984;29:393-395.

71. Arne J. Contribution a l'etude des oligomeres procyanidoliques: endotelon dans la retinopathie diabetique (a propose de 30 obervations). Gazette Medicale de France 1982;89:3810-3814.

72. Verin M, Vildy A, Maurin J. Retinopathies et OPC. Bordeaux Med 1978;11:1467-1474.

73. Fromantin M. Les oligomeres procyanidoliques dans le tratement de la fragilite capillaire et de la retinopathie chez les diabetiques. MedInt 1981;16:432-434.

74. Corbe C, Boissin J, Siou A. Sens lumineux et circulation chorioretinienne. Etude de l'effet des OPC (endotelon). J Fr Ophthalmol 1988;11:453-460.

75. Hughes-Formella B, Wunderlich O, Williams R. Anti-Inflammatory and Skin-Hydrating Properties of a Dietary Supplement and Topical Formulations Containing Oligomeric Proanthocyanidins. Skin Pharmacology and Physiology 2007;20:43-48.

76. de Haan CHA, van Dielen FMH, Houben AJHM, et al. Peripheral blood flow and noradrenaline responsiveness: the effect of physiological hyperinsulinemia. Cardiovasc Res 1997;34:192-198.

77. Houben AJHM, Kroon AA, de Haan CHA, Fuss-Lejeune MJMJ, de Leeuw PW. Quinaprilat-Induced Vasodilatation in Forearm Vasculature of Patients with Essential Hypertension: Comparison with Enalaprilat. Cardiovascular Drugs and Therapy 2000;14:657-663.

78. Houben AJHM, Kruseman ACN, Bouhouch E, Slaaf DW, Schaper NC. Peripheral macro-and microcirculation in short-term insulin-dependent diabetes mellitus: the role of prostaglandins in early haemodynamic changes. European Journal of Clinical Investigation 1993;23:662-667.

79. Houben AJHM, Schaper NC, Haan CHA, et al. The effects of 7-hour local hyperglycaemia on forearm macro and microcirculatory blood flow and vascular reactivity in healthy man. Diabetologia 1994;37:750-756.

80. Huvers FC, de Leeuw PW, de Haan CHA, Houben AJHM, Buijs C, Schaper NC. The enhanced pressor response in type 2 diabetes is not based upon a generalized increase in vascular responsiveness. Cardiovasc Res 1998;38:206-214.

81. Huvers FC, Schaper NC, Houben AJHM, et al. Impaired arterial but not venous responsiveness to nitroglycerin in non-insulin-dependent diabetes mellitus. European Journal of Clinical Investigation 1997;27:360-365.

82. van der Zander K, Houben AJHM, Kroon AA, de Leeuw PW. Effects of brain natriuretic peptide on forearm vasculature: comparison with atrial natriuretic peptide. Cardiovasc Res 1999;44:595-600.

83. van der Zander K, Houben AJHM, Kroon AA, De Mey JGR, Smits PABM, de Leeuw PW. Nitric oxide and potassium channels are involved in brain natriuretic peptide induced vasodilatation in man. Journal of Hypertension 2002;20:493-499.

84. Turner J, Belch JJF, Khan F. Current Concepts in Assessment of Microvascular Endothelial Function Using Laser Doppler Imaging and Iontophoresis. Trends in Cardiovascular Medicine 2008;18:109-116.

85. Wang J-S, Yang CF, Liaw M-Y, Wong M-K. Suppressed cutaneous endothelial vascular control and hemodynamic changes in paretic extremities with edema in the extremities of patients with hemiplegia. Archives of Physical Medicine and Rehabilitation 2002;83:1017-1023.

86. Lambert J, Aarsen M, Donker AJM, Stehouwer CDA. Endothelium-Dependent and -Independent Vasodilation of Large Arteries in Normoalbuminuric Insulin-Dependent Diabetes Mellitus. Arterioscler Thromb Vasc Biol 1996;16:705-711.

87. Corretti MC, Anderson TJ, Benjamin EJ, et al. Guidelines for the ultrasound assessment of endothelial-dependent flow-mediated vasodilation of the brachial artery: A report of the International Brachial Artery Reactivity Task Force. J Am Coll Cardiol 2002;39:257-265.

88. Pyke KE, Hartnett JA, Tschakovsky ME. Are the dynamic response characteristics of brachial artery flow-mediated dilation sensitive to the magnitude of increase in shear stimulus? J Appl Physiol 2008;105:282-292.
